# Alkaloids in Tibetan Medicine *Corydalis conspersa* Maxim. and Their Hepatoprotective Effect Against Acute Liver Injury

**DOI:** 10.3390/molecules30102127

**Published:** 2025-05-11

**Authors:** Qiu Wang, Yingrui Jin, Fangyan Fan, Xueting Feng, Xuemei Yin, Xiaoling Wang, Zangjia Geng

**Affiliations:** College of Pharmacy and Food, Southwest Minzu University, Chengdu 610041, China; 221008002019@stu.swun.edu.cn (Q.W.); 231008002025@stu.swun.edu.cn (Y.J.); 202131803011@stu.swun.edu.cn (F.F.) 80300225@swun.edu.cn (X.F.); xuemeiyin@swun.edu.cn (X.Y.)

**Keywords:** *Corydalis conspersa* Maxim., alkaloid, structural identification, Tibetan medicine, acute liver injury

## Abstract

The aim of the present study was to investigate the alkaloids of Tibetan medicine *Corydalis conspersa* Maxim. and their hepatoprotective effect against carbon tetrachloride (CCl_4_)-induced acute liver injury (ALI). The ethanol extract of this herbal medicine was subjected to a phytochemical study. Network pharmacology (NP) and molecular docking were used to predict the active constituents and mechanism of action against ALI. Seven alkaloid components were isolated and identified from this herb medicine, including acetylcorynoline (**1**, ACE), corynoline (**2**), scoulerine (**3**), protopine (**4**), bulbocapnine (**5**, BBC), palmatine (**6**), and isocorydine (**7**, ISO), among which compounds **1**, **3**, and **5** were isolated from this plant for the first time. Pharmacological experiments have shown that compounds **1**, **5**, **7**, and the total alkaloids (TTA) of the plant exhibit good improvement effects on CCl_4_-induced ALI in mice. NP and molecular docking predicted that their mechanism of action may be related to targets such as STAT3, SRC, EGFR, PIK3CA, and HSP90AA1. These research findings provide a theoretical basis for the development of the medicinal value of Tibetan medicine *Corydalis conspersa* Maxim.

## 1. Introduction

Plants have a long history of being used as medicine. In the Xia Dynasty of China, the first treaty on pathology/pharmacology emerged, including more than 360 so-called prescriptions/drugs with antipyretic, emetic, diuretic, and sedative effects, and contained various chemical substances such as iron, arsenic, cinnabar, and saltpeter [1]. The Tibetan medicine *Corydalis conspersa* Maxim. is a perennial herbaceous plant of the genus *Corydalis* in the family Poppyaceae. It is mainly distributed in the west and northwest of China, including Gansu, Qinghai, Sichuan, and Xizang, at an altitude of 4000 to 5000 m [2]. This herbal medicine belongs to the category of cold medicines and is referred to as “DongRiSiWa” in the Tibetan medical classic “*Jingzhu materia medica*”. This book records that this plant was used for the treatment of plague, burns, chiba disease (heat syndrome), dysentery, etc. It was also used in prescriptions to treat hypertension and rabies [3]. Tibetan medicine preparations such as Shibawei Dangshen Pills, Renqing Changjue, Sanweiqunchenmin Powder, Qiwei Wenyi Powder, and Dahongheipeng Pills all contain this medicinal material [3,4]. Modern pharmacological studies showed this plant has various activities, such as liver protection, anti-cancer and anti-tumor effects, antibacterial, anti-inflammatory, and analgesic properties, and antioxidant activity [4]. Studying the material basis and pharmacological activities of Tibetan medicine is helpful to understand the cultural connotations of Tibetan medicine, promote the improvement of local medical service level, and promote the local economic development of local medicinal plants.

Currently, there are few studies on the chemical constituents of *Corydalis conspersa* Maxim. both domestically and internationally. Que Sheng et al. isolated 11 chemical components, including berberine, palmatine, corynoline, and columbamine [5], and then Wu Jiang et al. profiled 10 alkaloids using High Performance Liquid Chromatography-Mass Spectrometry [6], but the data were insufficient to determine their precise structures. In addition, Que Sheng et al. profiled 55 volatile components using supercritical carbon dioxide extraction combined with Gas Chromatography-Mass Spectrometry [7]. The above studies indicate that the chemical constituents of this plant are highly complex, and there are still numerous unknown compounds yet to be discovered. The pathogenesis of ALI involves various physiological processes, such as oxidative stress and inflammation release [8]. Alkaloids are natural antioxidants. Modern pharmacological studies have shown that antioxidants can clear reactive oxygen species and reduce oxidative damage to DNA and lipids by regulating the hemeoxygenase-1 and NF-E2-related factor 2 pathways. This coincides with the mechanism of CCl_4_-induced hepatotoxicity in mice [9,10]. NP is a new discipline that is based on a vast database and explores the mechanisms of diseases and drugs within the context of larger biological networks [11]. The integration of NP with biological experiments can offer a novel approach for exploring the potential therapeutic mechanisms of Tibetan medicine against diseases [12].

Therefore, this study utilized solvent extraction and silica gel column chromatography to isolate and identify the alkaloids in Tibetan medicine *Corydalis conspersa* Maxim. NP and molecular docking techniques were employed to predict the hepatoprotective mechanism of those alkaloids. The hepatoprotective effects of the main single alkaloids and total alkaloids were determined using a CCl_4_-induced ALI mouse model. According to literature reports, Kunming (KM) mice are a closed colony of mice with high genetic heterogeneity, and they exhibit significant responses to CCl_4_-induced ALI. Therefore, KM mice were selected as the model animals [13,14,15,16]. This study aimed to provide a pharmaceutical material basis for the further research and development of *Corydalis conspersa* Maxim.

## 2. Results

### 2.1. Alkaloids in Corydalis conspersa Maxim.

All the spectra of hydrogen, carbon and mass spectrometry were included in the supplementary materials (Figures S1–S21). The results were listed as follows Figure 1.

**Figure 1:**
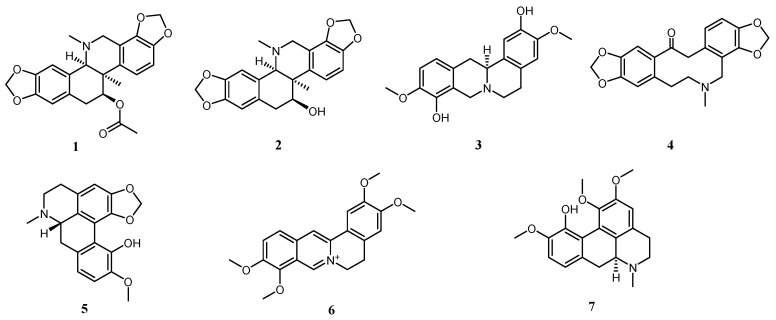
Structure of compounds **1**~**7**.

Compound **1**: White massive crystals (acetone). The molecular formula was determined to be C_23_H_23_NO_6_. HR-ESI-MS *m*/*z*: 408.14401 [M − H]^+^. ^1^H-NMR (400 MHz, CDCl_3_) *δ* 7.04 (d, *J* = 8.3 Hz, 1H, H-10), 6.93 (s, 1H, H-4), 6.74 (d, *J* = 8.3 Hz, 1H, H-9), 6.57 (s, 1H, H-1), 5.93–6.05 (4H, m, 2× -O-CH_2_-O-, 2, 3-, 7, 8-), 5.26 (dd, *J* = 8.6, 6.4 Hz, 1H, H-11), 3.95 (d, *J* = 16.0 Hz, 1H, H-6), 3.58 (d, *J* = 13.8 Hz, 2H, H-6, H-14), 3.02 (dd, *J* = 15.3, 8.8 Hz, 1H, *H-12α*), 2.89 (dd, *J* = 15.3, 6.2 Hz, 1H, *H-12β*), 2.53 (s, 3H, 5-NCH_3_), 1.92 (s, 3H, COCH_3_), 1.32 (s, 3H, 13-CH_3_); ^13^C-NMR (150 MHz) *δ* 106.44 (C-1), 146.23 (C-2), 146.87 (C-3), 100.95 (2-O-CH_2_-O-3), 109.64 (C-4), 130.14 (C-4a), 49.68 (C-6), 117.73 (C-6a), 143.10 (C-7), 144.81 (C-8), 101.14 (7-O-CH_2_-O-8), 108.55 (C-9), 120.50 (C-10), 133.15 (C-10a), 75.61 (C-11), 32.94 (C-12), 127.65 (C-12a), 42.60 (C-13), 70.31 (C-14), 43.86 (C-5-NCH_3_), 27.99 (CH_3_-13), 170.72, 21.33 (OAc). The above data match those of acetylcorynoline reported in the literature [17]. Thus, compound **1** is confirmed to be acetylcorynoline. Its structure is shown in Figure 1 (**1**).

Compound **2**: Colorless bulk crystals (acetone). The molecular formula was determined to be C_21_H_21_NO_5_. HR-ESI-MS *m*/*z*: 368.14920 [M + H]^+^. ^1^H-NMR (400 MHz, CDCl_3_) *δ* 6.98 (dd, *J* = 8.2, 1.5 Hz, 1H, H-10), 6.85 (d, *J* = 8.2 Hz, 1H, H-9), 6.70 (d, *J* = 6.6 Hz, 2H, H-4,H-1), 6.07–5.97 (m, 4H, 2× -O-CH_2_-O-, 2, 3-, 7, 8-), 4.09, 3.50 (d, *J* = 15.3 Hz, 2H, H-6), 4.01 (dd, *J* = 4.6, 2.1 Hz, 1H, H-11), 3.36 (d, *J* = 2.1 Hz, 1H, H-14), 3.26–3.09 (m, 2H, H*-12α*, *H-12β*), 2.27 (d, *J* = 1.6 Hz, 3H, 5-NCH_3_), 1.20 (d, *J* = 1.6 Hz, 3H, CH_3_-13); ^13^C-NMR (150 MHz) *δ* 107.73 (C-1), 145.33 (C-2), 148.04 (C-3), 101.06 (2-O-CH_2_-O-3), 112.75 (C-4), 127.93 (C-4a), 54.32 (C-6), 116.76 (C-6a), 142.76 (C-7), 145.11 (C-8), 101.39 (7-O-CH_2_-O-8), 109.45 (C-9), 118.66 (C-10), 136.02 (C-10a), 76.14 (C-11), 36.74 (C-12), 125.13 (C-12a), 40.83 (C-13), 69.81 (C-14), 43.26 (C-5-NCH_3_), 23.43 (CH_3_-13). The above data match those of corynoline reported in the literature [17]. Thus, compound **2** is confirmed to be corynoline. Its structure is shown in Figure 1 (**2**).

Compound **3**: Reddish brown amorphous powder. The molecular formula was determined to be C_19_H_21_NO_4_. HR-ESI-MS *m*/*z*: 328.15372 [M + H]^+^. ^1^H-NMR (400 MHz, CDCl_3_) *δ* 6.87 (s, 1H, H-1), 6.81–6.75 (m, 1H, H-11), 6.72 (d, *J* = 8.3 Hz, 1H, H-12), 6.65 (s, 1H, H-4), 4.29 (d, *J* = 15.5 Hz, 1H, *H-8β*), 4.04- 3.70 (dd, *J* = 3.7, 1.7 Hz, 6H, 3, 10-OCH_3_), 3.57 (t, *J* = 13.2 Hz, 2H, *H-8α*, 14), 3.33–3.22 (m, 2H, *H-6β*, *H-13β*), 3.22–3.13 (m, 1H, H-*5β*), 2.87 (dd, *J* = 15.9, 11.4 Hz, 1H, *H-13α*), 2.71 (t, *J* = 8.4 Hz, 2H, *H-6α*, *H-5α*); ^13^C-NMR (150 MHz) *δ* 109.08 (C-1), 145.17 (C-2), 144.00 (C-3), 56.24 (3-OCH_3_), 111.43 (C-4), 130.55 (C-4a), 29.13 (C-5), 51.60 (C-6), 53.51 (C-8), 120.92 (C-8a), 141.56 (C-9), 144.08 (C-10), 55.99 (10-OCH_3_), 110.68 (C-11), 119.44 (C-12), 128.09 (C-12a), 36.24 (C-13), 59.25 (C-14), 126.06 (C-14a). The above data match those of scoulerine reported in the literature [18]. Thus, compound **3** is confirmed to be scoulerine. Its structure is shown in Figure 1 (**3**).

Compound **4**: White amorphous powder. The molecular formula was determined to be C_20_H_19_NO_5_. HR-ESI-MS *m*/*z*: 354.13315 [M + H]^+^. ^1^H-NMR (400 MHz, CDCl_3_) *δ* 6.95 (d, *J* = 1.5 Hz, 1H, H-1), 6.77–6.67 (m, 3H, H-4, 11, 12), 5.99 (dd, *J* = 10.3, 1.5 Hz, 4H, H-15, 16), 1.97 (s, 3H, 7-NCH_3_); ^13^C-NMR (150 MHz) *δ* 41.60 (N-CH_3_), 108.23 (C-1), 146.39 (C-2), 148.10 (C-3), 101.30 (2,3-OCH_2_O-), 110.54 (C-4), 132.73 (C-4a), 31.78 (C-5), 57.85 (C-6), 50.96 (C-8), 117.83 (C-8a), 146.00 (C-9), 146.09 (C-10), 100.97 (9,10-OCH_2_O-), 106.85 (C-11), 125.11 (C-12), 128.98 (C-12a), 46.43 (C-13), 179.84 (C-14), 136.09 (C-14a). The above data match those of protopine reported in the literature [19]. Thus, compound **4** is confirmed to be protopine. Its structure is shown in Figure 1 (**4**).

Compound **5**: Brownish yellow needle-like crystals (acetone). The molecular formula was determined to be C_19_H_19_NO_4_. HR-ESI-MS *m*/*z*: 326.13838 [M + H]^+^. ^1^H-NMR (400 MHz, CDCl_3_) *δ* 7.09 (s, 1H, -OH), 6.88 (d, *J* = 1.8 Hz, 2H, H-9, 10), 6.69 (s, 1H, H-4), 6.15, 6.00 (q, d *J* = 1.2, 1.6 Hz, 2H, -OCH_2_O-), 3.98–3.93 (m, 3H, -OCH_3_), 3.25–2.96 (m, 4H. H-5, 6, 8), 2.59 (d, *J* = 1.7 Hz, 3H, N-CH_3_); ^13^C-NMR (150 MHz) *δ* 44.02 (N-CH_3_), 56.26 (-OCH_3_), 100.36 (-OCH_2_O-), 114.37 (C-12a), 118.52 (C-1), 129.73 (C-1a), 140.60 (C-2), 146.07 (C-3), 107.76 (C-4), 127.42 (C-4a), 29.35 (C-5), 53.05 (C-6), 62.82 (C-7a), 35.39 (C-8), 128.88 (C-8a), 119.39 (C-9), 110.90 (C-10), 148.38 (C-11), 142.94 (C-12). The above data match those of bulbocapnine reported in the literature [20]. Thus, compound **5** is confirmed to be bulbocapnine. Its structure is shown in Figure 1 (**5**).

Compound **6**: Yellow powder. The molecular formula was determined to be C_21_H_22_N^+^O_4_. HR-ESI-MS *m*/*z*: 352.15391 [M + H]^+^. ^1^H-NMR (600 MHz, CD_3_OD) *δ* 9.76 (s, 1H, H-8), 8.81 (s, 1H, H-13), 8.12 (d, *J* = 9.1 Hz, 1H, H-11), 8.02 (d, *J* = 9.0 Hz, 1H, H-12), 7.67 (s, 1H, H-1), 7.05 (s, 1H, H-4), 4.94 (t, *J* = 6.4 Hz, 2H, H-6), 4.21 (s, 3H, 9-OCH_3_), 4.11 (s, 3H, 10-OCH_3_), 3.99 (s, 3H, 2-OCH_3_), 3.94 (s, 3H, 3-OCH_3_), 3.28 (t, *J* = 6.3 Hz, 2H, H-5); ^13^C-NMR (200 MHz) *δ* 108.68 (C-1), 149.34 (C-2), 152.44 (C-3), 110.89 (C-4), 128.72 (C-4a), 26.41 (C-5), 56.28 (C-6), 144.99 (C-8), 119.08 (C-8a), 150.46 (C-9), 144.38 (C-10), 119.89 (C-11), 123.05 (C-12), 133.91 (C-12a), 126.81 (C-13), 138.47 (C-14), 121.91 (C-14a), 61.13 (-OCH_3_), 55.97 (-OCH_3_), 55.63 (-OCH_3_), 55.29 (-OCH_3_). The above data match those of palmatine reported in the literature [17]. Thus, compound **6** is confirmed to be palmatine. Its structure is shown in Figure 1 (**6**).

Compound **7** (C_20_H_23_NO_4_): Yellow powder. The molecular formula was determined to be C_20_H_23_NO_4_. HR-ESI-MS *m*/*z*: 342.16975 [M + H]^+^. ^1^H-NMR (600 MHz, CDCl_3_) *δ* 8.81 (s, 1H, 11-OH), 6.85 (q, *J* = 8.1 Hz, 2H, H-8, H-9), 6.70 (s, 1H, H-3), 3.91 (d, *J* = 5.9 Hz, 6H, 10-OCH_3_, 2-OCH_3_), 3.71 (s. 3H, 1-OCH_3_), 3.22 (s, 1H, H-6a), 3.05 (dd, *J* = 13.0, 3.5 Hz, 2H, H-4), 2.92 (d, *J* = 12.6 Hz, 1H, H-7a), 2.72 (dd, *J* = 16.6, 3.7 Hz, 1H, H-7b), 2.56 (s, 3H, N-CH_3_), 2.51 (s, 2H, H-5); ^13^C-NMR (200 MHz) *δ* 142.28 (C-1), 151.40 (C-2), 111.19 (C-3), 130.14 (C-3a), 125.99 (C-3b), 29.77 (C-4), 52.76 (C-5), 62.92 (C-6a), 35.82 (C-7), 130.20 (C-7a), 119.02 (C-8), 109.83 (C-9), 149.53 (C-10), 144.09 (C-11), 120.11 (C-11a), 129.42 (C-11b), 62.09 (1-OCH_3_), 55.93 (2-OCH_3_), 56.20 (10-OCH_3_), 43.94 (N-CH_3_). The above data match those of isocorydine reported in the literature [21]. Thus, compound **7** is confirmed to be isocorydine. Its structure is shown in Figure 1 (**7**).

### 2.2. NP Prediction Results

#### 2.2.1. Possible Targets of ALI Affected by the Chemical Components of *Corydalis conspersa* Maxim.

Based on the collection of 21 chemical components from *Corydalis conspersa* Maxim. in databases such as China National Knowledge Infrastructure, 16 pharmaceutical components were identified using the Swiss ADME platform. Through the Swiss Target Prediction database, target prediction was conducted for these 16 pharmaceutical components, resulting in 234 potential targets after removing duplicates. Using the Gene Cards database, ALI targets were predicted, and after filtering and removing duplicates based on a correlation score greater than 20, 1136 disease-related targets were obtained. The predicted targets were then input into the Venn platform to intersect and obtain a total of 70 disease-compound targets.

#### 2.2.2. Construction of Protein–Protein Interaction (PPI) Network

**Figure 2:**
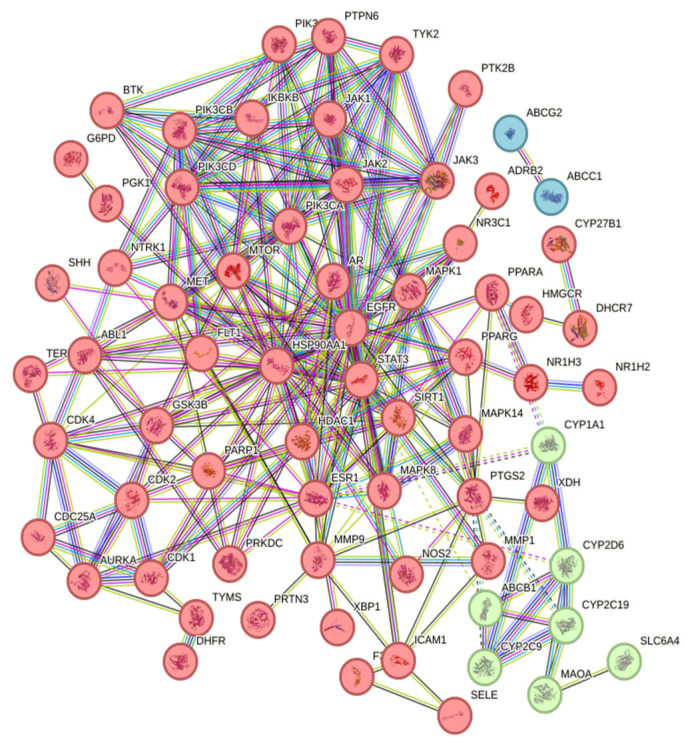
PPI network diagram of intersecting targets. Note: Red represents gene clusters that can regulate tyrosine protein kinase receptor-mediated signaling pathways; Green represents the gene cluster that can regulate the catabolism pathway of cytochrome P450 in xenobiotic organisms; Blue represents the gene cluster that can regulate the anti-folate resistance pathway.

**Figure 3:**
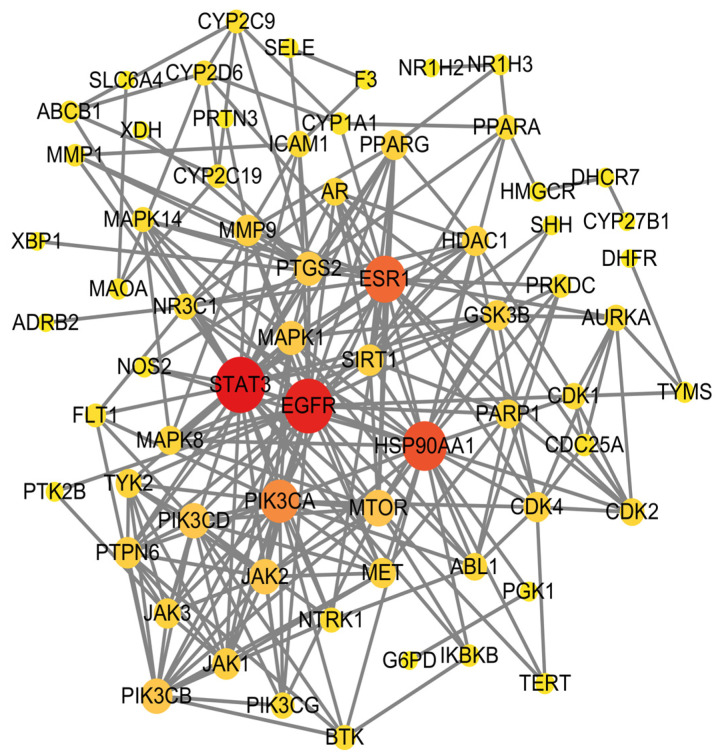
PPI network diagram of core targets. Note: The larger the target shape and the darker the color, the greater the degree value.

A total of 70 intersecting targets were imported into the STRING database to construct a PPI network, as shown in Figure 2. The network was optimized using Cytoscape 3.10.2 software, and the top five core targets were identified based on their degree values. The larger the degree value of a target, the darker its color, indicating that it was a core target, as shown in Figure 3. The core targets were STAT3, PIK3CA, EGFR, ESR1, and HSP90AA1, as listed in Table 1.

#### 2.2.3. Construction of Drug-Active Ingredient-Target Network Diagram

**Figure 4:**
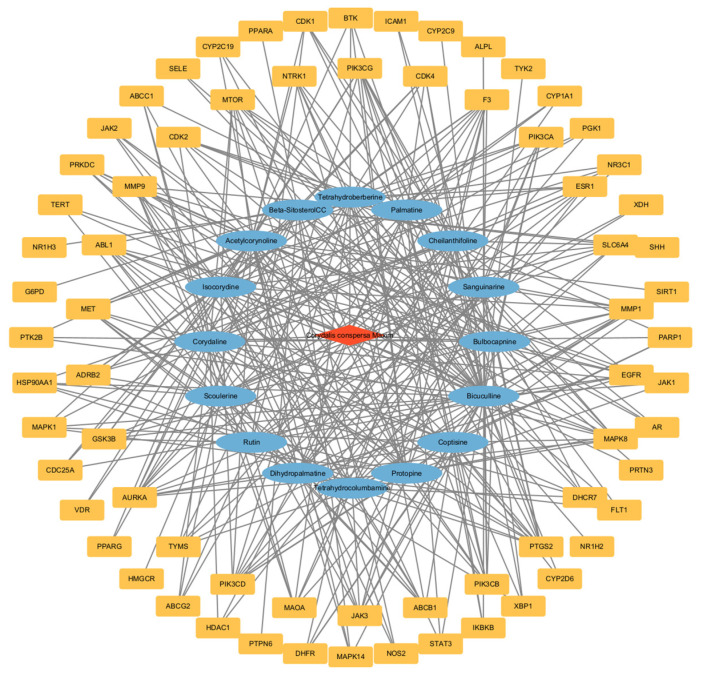
Drug-active ingredient-target network diagram. Note: The red diamond represents *Corydalis conspersa* Maxim., the blue oval represents the chemical composition, and the yellow square represents the intersection target.

The active components of *Corydalis conspersa* Maxim. and their intersecting targets were imported into Cytoscape 3.10.2 software to construct a drug-active component–target network diagram. This diagram consisted of 87 nodes and 352 edges. In the network diagram, blue represents active components, yellow represents targets, and red represents drugs. By constructing this diagram for the treatment of ALI with *Corydalis conspersa* Maxim., it was further revealed that *Corydalis conspersa* Maxim. exerts effects on ALI through multiple targets and pathways. The results are shown in Figure 4. The network analyzer tool Cytoscape 3.10.2 was used to calculate the network topological parameters, and the top five chemical components with the highest degree values were selected as core active components, as shown in Table 2.

#### 2.2.4. Gene Ontology (GO) and Kyoto Encyclopedia of Genes and Genomes (KEGG) Enrichment Analysis

**Figure 5:**
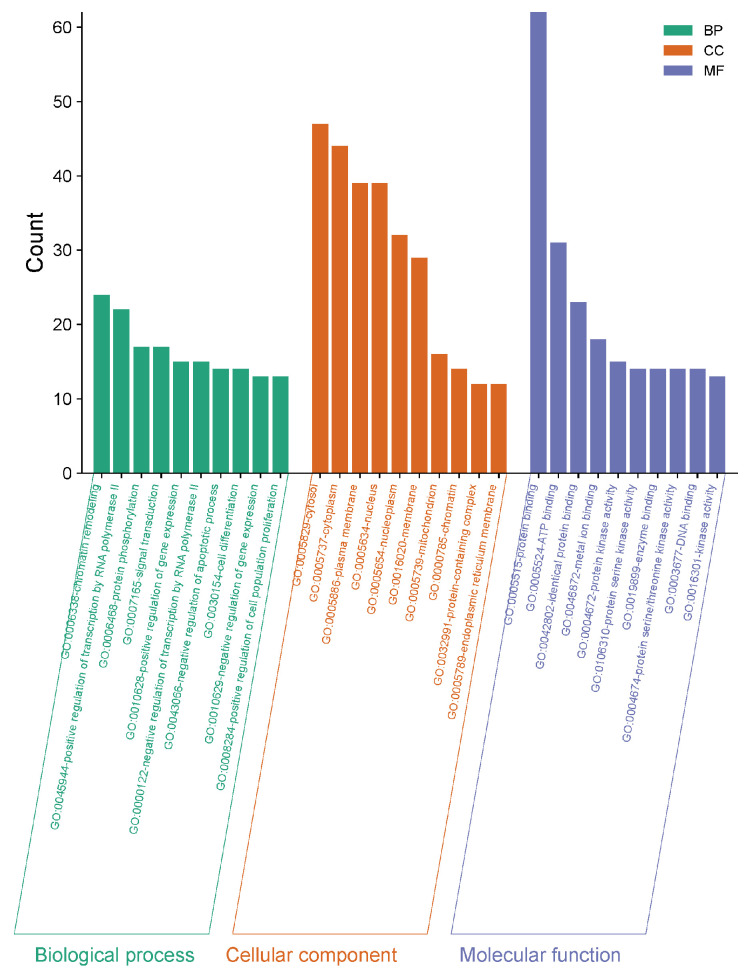
GO function analysis.

**Figure 6:**
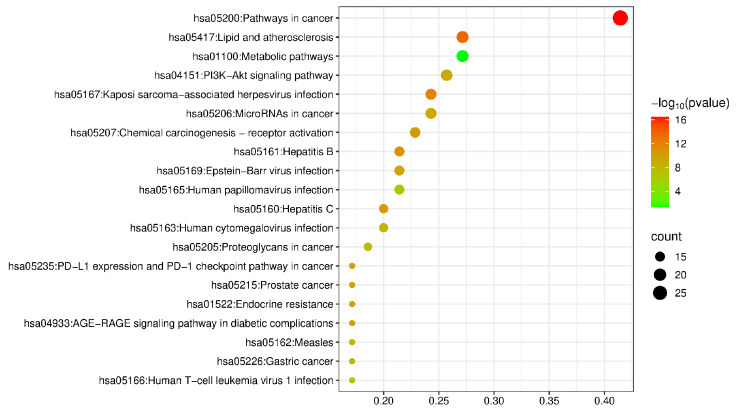
KEGG pathway enrichment analysis.

According to the method described in Section 4.2.2, GO enrichment analysis was conducted on 70 common targets. The results showed that there were 164 enriched biological process (BP) entries, which were related to chromatin remodeling, protein phosphorylation reactions, and regulation of the apoptosis process; 284 enriched cellular component (CC) entries, which were related to the plasma membrane, cytoplasm, cytosol, and nucleus; and 218 enriched molecular function (MF) entries, which were related to ATP binding, protein kinase activity, and protein-binding receptor activity. KEGG pathway enrichment analysis yielded a total of 563 pathways, mainly involving the PI3K-Akt signaling pathway, lipid and atherosclerosis, hepatitis B, hepatitis C, and other pathways. The results are shown in Figure 5 and Figure 6.

#### 2.2.5. Molecular Docking Verification Results

**Figure 7:**
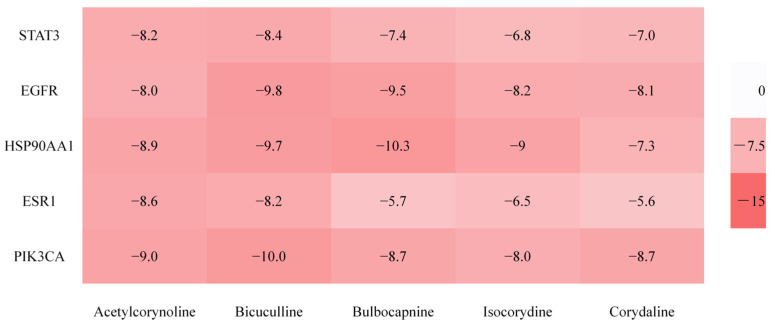
Molecular docking verification results. Note: The number represents the binding energy (kcal/mol), the horizontal direction represents the active ingredient, and the vertical direction represents the core target point.

**Figure 8:**
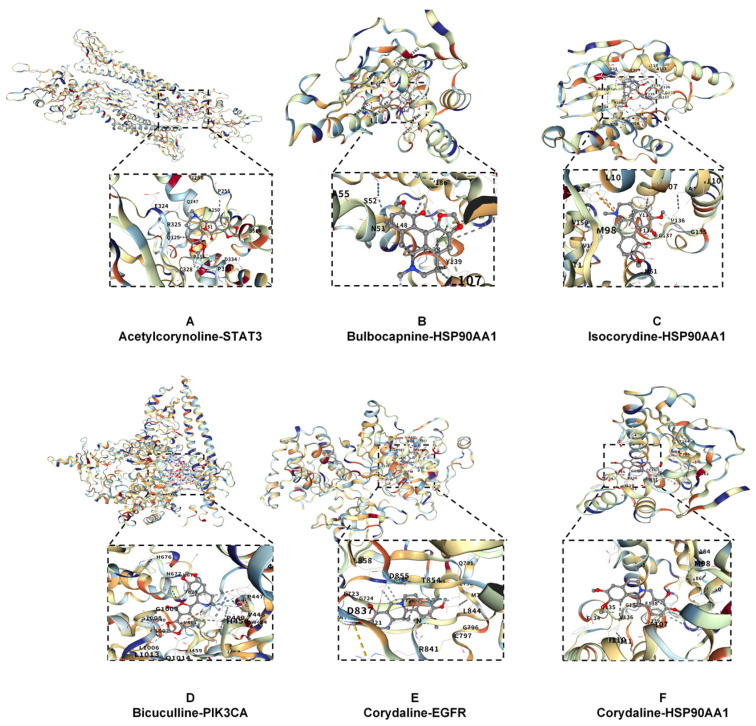
Molecular docking verification results.

The binding energy (kcal/mol) between the active components of *Corydalis conspersa* Maxim. and its core targets were evaluated according to the method described in Section 4.2.2. The binding energy is shown in Figure 7, where a stronger binding energy is indicated by a redder color. When the binding energy is less than −5 kcal/mol, it indicates a good binding between the target and the component. The lower the binding energy, the more stable the binding between the component and the target. The results in Figure 7 show values less than −5 kcal/mol, suggesting that the active components of *Corydalis conspersa* Maxim. bind tightly to their targets. This indicates that the drug exerts therapeutic effects on ALI by regulating STAT3, SRC, EGFR, ESR1, HSP90AA1, etc., through active components such as acetylcorydaline, bulbocapnine, corydaline, isocorydaline, and bicuculline. Among them, the binding energy between bulbocapnine and HSP90AA1 was −10.3 kcal/mol, indicating the strongest binding ability. Similar results can be deduced. This result was consistent with NP predictions. Six randomly selected examples of binding sites are shown in Figure 8.

We ultimately identified 21 active components of *Corydalis conspersa* Maxim. and 70 intersecting targets between drugs and diseases through relevant databases. By integrating the “drug-active component–target” network, the key active components of *Corydalis conspersa* Maxim. for treating ALI were determined to be bicuculline, acetylcorynoline, corynoline, isocorydine, and bulbocapnine. Through constructing a PPI network, five potential targets for *Corydalis conspersa* Maxim. in the treatment of ALI were identified: STAT3, PIK3CA, EGFR, ESR1, and HSP90AA1.

### 2.3. Experimental Results of Hepatoprotective Effect

#### 2.3.1. Changes in Serum Biochemical Indicators of Mice

Based on the variations in serum aspartate aminotransferase (AST), alanine aminotransferase (ALT), and alkaline phosphatase (ALP) levels among various groups of mice (refer to Table 3), it is evident that these levels in CCl_4_-treated mice were notably elevated compared to those in the control (CON) group (*p* < 0.001). However, in the ACE, BBC, and ISO groups administered at doses of 5, 10, and 20 mg/kg, respectively, the levels in mice were reduced. When the dose reached 10 mg/kg, the levels were significantly reduced. The reduction levels in each group were positively correlated with the dose, and the differences were statistically significant compared to the CCl_4_ group (*p* < 0.05, *p* < 0.01, or *p* < 0.001). Additionally, the levels in mice across all TTA dose groups were significantly lowered. Similarly, the levels in mice from the positive silymarin (SIL) group were notably reduced, exhibiting statistically significant differences compared to the CCl_4_ group (*p* < 0.001), as outlined in Table 3.

#### 2.3.2. Changes in C-Reactive Protein (CRP) Levels in Mouse Liver

The CRP activity in the liver of the CCl_4_ group was notably higher than that of the CON group (*p* < 0.001). Compared to those in the CCl_4_ group, the CRP levels in the ACE, BBC, ISO, and TTA groups were all reduced, with statistically significant differences except for the ISO 5 mg/kg group (*p* < 0.05, *p* < 0.01, or *p* < 0.001). The extent of CRP reduction positively correlated with the dosage. Compared to the CCl_4_ group, the CRP activity in the SIL group was significantly reduced, with a statistically significant difference (*p* < 0.001), as presented in Table 4.

Based on the levels of AST, ALT, and ALP in serum and CRP in the liver tissue of mice, when compared to the CON group, the levels of AST, ALT, ALP, and CRP in the CCl_4_ group were significantly elevated, indicating the successful establishment of the ALI model. The hepatoprotective effects of the low- and medium-dose groups of ACE and BBC on CCl_4_-induced ALI in mice were inferior to those of the SIL group, but the high-dose group exhibited a hepatoprotective effect close to that of the SIL group. The biological activity of the ISO group at the same dose was weaker compared to the ACE and BBC groups. All dose groups of TTA demonstrated good hepatoprotective effects. Therefore, the alkaloids obtained from Tibetan medicine *Corydalis conspersa* Maxim. exhibited significant hepatoprotective activity at the molecular level, especially compounds ACE and BBC, which exhibited excellent hepatoprotective effects.

#### 2.3.3. Results of H&E-Stained Sections

**Figure 9:**
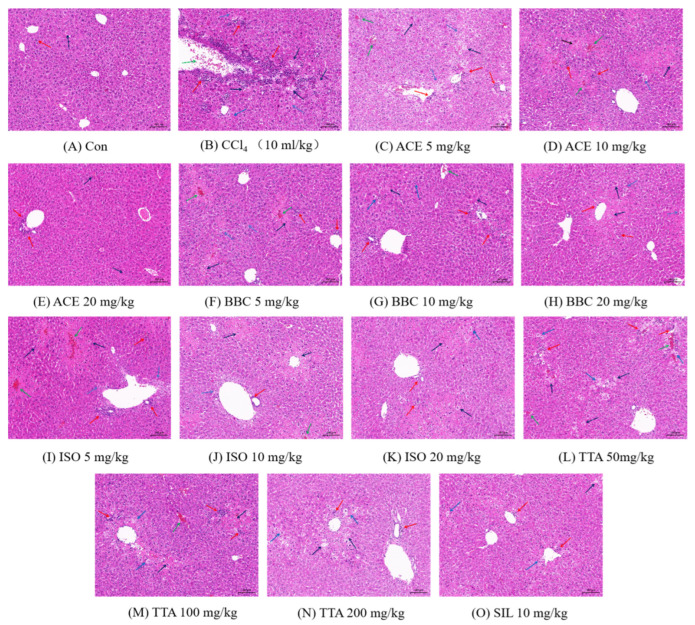
Effects of extract on CCl_4_-induced liver histopathological changes (200×). Note: (**A**) is the CON group, (**B**) is the CCl_4_ group, and (**C**–**O**) are the drug administration groups. The number refers to the dosage. The red arrow indicates inflammatory cell infiltration, the black arrow indicates hepatocyte necrosis, the blue arrow indicates hepatocyte swelling, and the green arrow indicates congestion in the central vein.

The H&E staining results of the mouse liver are shown in Figure 9 below. In the CON group (Figure 9A), the liver lobule structure is clear, the hepatocytes are arranged regularly, the morphology is normal, and the liver cords are arranged radially. Only a small number of samples showed a small amount of inflammatory cell infiltration, which is a normal physiological phenomenon. In the CCl_4_ group (Figure 9B), the hepatocytes are arranged in disorder, and the intercellular spaces are enlarged. There is a large amount of inflammatory cell infiltration and dead hepatocytes, mainly concentrated near the central vein, with central venous congestion and a fragmented liver lobule structure. This is a typical manifestation of acute liver toxicity, indicating successful modeling. Compared with the CCl_4_ group, the test groups (Figure 9C–O, portal vein area, pericentral vein area, and other regions) exhibited a significant reduction in the number of hepatocyte necroses, narrowed intercellular spaces, decreased inflammatory cell infiltration, and alleviated congestion in the central vein. Although no notable improvement was observed in the low-dose group, the degree of liver injury significantly decreased with increasing dosage. The above results showed that the test groups have a significant liver repair effect compared with the CCl_4_ group, in a dose-dependent manner. Compared with the test groups, the SIL group showed no significant hepatocyte necrosis, smaller cell gaps, a significantly reduced number of inflammatory cells, and no significant central venous congestion, demonstrating better liver injury repair effects.

## 3. Discussion

The liver is a major detoxification and metabolism organ in the human body and has an irreplaceable role in people’s daily life. The pathogenesis of ALI is often due to inflammatory reactions and lipid peroxidation [22]. CCl_4_ is a hepatotoxic substance commonly used in the production of experimental models of acute liver injury, and its mechanism of action is complex. Modern pharmacological studies have revealed this mechanism of action of CCl_4_, which is activated by the metabolism of cytochrome P450 enzymes after its entry into hepatocytes, generating trichloromethyl radicals and trichloromethyl peroxide radicals. These free radicals will covalently bind to intracellular phospholipids, triggering lipid peroxidation, thus damaging the structure and function of the cell membrane [23]. After the structure and function of the hepatocyte membrane are damaged, the transaminases ALP, ALT, and AST in the cells overflow, causing the content of ALP, ALT, and AST in the serum to increase. Therefore, the elevation of serum ALP, ALT, and AST is usually considered an important indicator of the severity of ALI [24].

ACE is one of the main active ingredients in *Corydalis conspersa* Maxim., and it has significant repairing effects on ALI induced by lipopolysaccharide/D-Galactosamine (LPS/D-GaIN) in mice. Fu Jun et al. used RNA sequencing combined with molecular docking technology to reveal that it can alleviate LPS/D-GaIN-induced ALI by acting on P65 and JNK receptors, and used quantitative Polymerase Chain Reaction technology to show that ACE may also exert anti-inflammatory effects by inhibiting key targets in the Toll-like receptor signaling pathway to alleviate alcohol liver disease. In addition, they conducted cell experiments to compare the levels of inflammatory factors in cells after ACE and LPS treatment, and used Reverse Transcription-quantitative Polymerase Chain Reaction technology to explore the expression of mRNA in cells. The results showed that ACE had anti-inflammatory effects and inhibited the expression of TLR4 and FOS mRNA in cells induced by LPS [25].

ACE, BBC, and ISO are all isoquinoline alkaloids with similar chemical structures, so their mechanisms of action may be the same or similar. We speculate that ACE, BBC, and ISO alleviate the inflammatory response CCl_4_-induced ALI in mice by suppressing the expression of inflammatory factors, thereby reducing ALI in mice. This study provides certain theoretical support for the research results of this article. The aforementioned modern pharmacological research provides theoretical support for our results.

## 4. Materials and Methods

### 4.1. Experimental Materials

#### 4.1.1. Experimental Medicinal Materials

The dried whole herb of *Corydalis conspersa* Maxim. was collected from Changdu City, Tibet Autonomous Region, and was identified as a perennial plant of the genus *Corydalis* in the family of Poppies by Dawa Zhuoma, Chief Pharmacist of the Tibet Autonomous Region Drug Administration. The specimen was deposited in the Herbarium of the College of Pharmacy and Food, Southwest Minzu University. The specimen number is 2023031503.

#### 4.1.2. Animals and Drugs

Eighty healthy male KM mice (SPF grade) were purchased from Chengdu Dashuo Laboratory Animal Co., Ltd. (Chengdu, China) (Batch No.: SCXK (Chuan) 2020-0030) and approved for use by the Academic Committee of Southwest Minzu University (No.: SMU-202401114). TTA was isolated from *Corydalis conspersa* Maxim. in our laboratory; ACE, BBC, and ISO (≥99.9%, Chengdu Push Biotechnology Co., Ltd., Chengdu, China); and SIL (≥99.9%, Shanghai Macklin Biochemical Technology Co., Ltd., Shanghai, China).

#### 4.1.3. Reagents and Instruments

The following reagents and instruments were used: methanol (MeOH), petroleum ether (PE), and ethyl acetate (EtOAc) (Analytical Reagent, Tianjin Fengchuan Chemical Reagent Co., Ltd., Tianjin, China); chloroform (CHCl_3_), concentrated hydrochloric acid (HCl, 37%), sodium hydroxide, ammonia (25–28%), xylene, and anhydrous ethanol (EtOH) (Analytical Reagent, Chengdu Chron Chemical Co., Ltd., Chengdu, China); neutral gum (Sinopharm Chemical Reagent Co., Ltd., Shanghai, China); H&E dye set (Leagene, Beijing, China); sliced paraffin wax (Shanghai Huayong Paraffin Co., Ltd., Shanghai, China); silica gel column chromatography packing (100–200 mesh and 200–300 mesh, Qingdao Ocean Chemical Co., Ltd., Qingdao, China); silica gel 60 packing (Merck, Darmstadt, Germany); silica gel GF_254_ thin-layer plate (Qingdao Ocean Chemical Co., Ltd. China), rotary evaporator (RE-2000A, Shanghai Yarong Biochemical Instrument Factory, Shanghai, China); low-temperature coolant circulating pump (DL-400, Zhengzhou Huicheng Science, Industry and Trade Co., Ltd., Zhengzhou, China); circulating water-type multi-purpose vacuum pump (SHB-III, Zhengzhou Great Wall Science, Industry and Trade Co., Ltd., Zhengzhou, China); Bruck AVANCE III 400 M Nuclear Magnetic Resonance Instrument (Bruker, Munich, Germany); Bruck AVANCE NEO 600 M Nuclear Magnetic Resonance Instrument (Bruker, Germany); Bruck SolanX 70 FT-MS High-Resolution Mass Spectrometry (Bruker, Germany); panoramic slice scanner PANNORAMIC SCAN (3DHISTECH, Budapest, Hungary); orthogonal white-light photomicrographic microscope (Primo Star, ZEISS, Oberkochen, Germany); and ALP, ALT, AST, and CRP kits (Myriad BioMedical Electronics Co., Ltd., Shenzhen, China).

### 4.2. Methods

#### 4.2.1. Extraction and Separation Methods

The dried and powdered whole herb plants (10.0 kg) were extracted with 95% EtOH (7 days × 3) at room temperature. The solvent was evaporated in vacuo to give a dark-brown extract (1.2 kg), which was then dispersed in HCl (0.5%, 3 L), stirred well, and then left overnight. After extracting the acidic aqueous solution with EtOAc (3 L), the aqueous phase was adjusted to pH 11–12 using a 20% NaOH aqueous solution. After thorough stirring, it was left to stand overnight. It was then extracted sequentially with equal volumes of PE and CHCl_3_ to obtain the PE fraction and CHCl_3_ fraction, respectively. The CHCl_3_ fraction was concentrated under reduced pressure to recover the solvent, yielding total alkaloids (46 g).

The separation was carried out by silica gel column chromatography (SGCC, 100–200 mesh) with the eluent of PE: EtOAc (10:1→0:1, *v*/*v*), and a total of 15 fractions (Fr.1–Fr.15) were obtained. Among them, Fr.4 (1.3 g) was concentrated and then recrystallized in acetone to give compound **1** (77.1 mg). Fr.5 (3.1 g) was applied to repeated SGCC (200–300 mesh) (PE/EtOAc, 10:1→0:1, *v*/*v*) to give 6 fractions (Fr.5.1–Fr.5.6), of which Fr.5.2 was concentrated to afford **2** (212.7 mg), and Fr.8 (2.3 g) was applied to repeated SGCC (PE/EtOAc, 5:1→0:1, *v*/*v*) to afford **3** (25.2 mg), **4** (244 mg), and **5** (282 mg); Fr.11 (3.2 g) was purified by chromatography using silica gel Si60, with CHCl_3_/ MeOH (15:1→0:1, *v*/*v*) in gradient elution to afford **6** (35.8 mg). Fr11.6 was purified using silica gel 60 chromatography with CHCl_3_/CH_3_OH (10:1→0:1, *v*/*v*) gradient elution to afford **7** (40.6 mg).

#### 4.2.2. Research Methods of NP

The methodology of this study follows [26].

Through preliminary experimental research and literature retrieval—using databases such as China National Knowledge Infrastructure and Public/Publisher MEDLINE, as well as relevant academic materials—serving as the primary data sources, a collection of chemical components was obtained to establish the component set of *Corydalis conspersa* Maxim. The Simplified Molecular Input Line Entry System structural formulas of drug components were obtained from the Public/Publisher MEDLINE database (https://pubchem.ncbi.nlm.nih.gov/). Using these obtained Simplified Molecular Input Line Entry System structural formulas, screening was conducted via the Swiss ADME platform, with the criteria of a “high” score for gastrointestinal absorption and at least two “yes” responses for drug likeness. The predicted targets of the screened drug components were analyzed on the Swiss Target Prediction platform (http://www.Swisstargetprediction.ch/index.php), and according to a probability* ≥ 0.1, the duplicate values were screened and removed to find the possible action targets of *Corydalis conspersa* Maxim.

Using the standard English term “Acute liver injury” as the search term, we conducted a search in the Gene Cards (https://www.genecards.org/) database to obtain ALI-related disease targets. These targets were then filtered based on a relevance score greater than 20.

The obtained target points of each chemical component and the ALI disease target points were imported into the bioinformatics and evolutionary genomics website (http://bioinformatics.psb.ugent.be/webtools/Venn/) to obtain the intersection target points between the chemical components and the ALI disease.

The key target genes were imported into the STRING (https://cn.string-db.org/) platform, the protein type was selected as “homosapiens”, and the minimum interaction domain value was set to ≥0.7. The intersection target PPI network was obtained, the PPI network diagram was imported into Cytoscape 3.10.2 software, and the “Network Analyzer” function in the software was utilized to perform network topology analysis. Then, core targets were screened based on the degree value.

The active components of *Corydalis conspersa* Maxim. and the intersecting targets were imported into Cytoscape 3.10.2 software to construct a drug-active component–target network diagram, and then key components were screened based on degree values.

The intersecting gene data were imported into the DAVID database (https://davidbioinformatics.nih.gov), the species was set to “Homo sapiens”, and GO function and KEGG pathway enrichment analyses were performed. GO includes information such as BP, MF, and CC. For GO analysis, the top 10 results were selected, while for KEGG analysis, the top 20 results were chosen. Additionally, the MicroBioinformatics website (http://www.bioinformatics.com.cn/) was utilized to draw GO analysis bar charts, and online KEGG enrichment analysis bubble charts were used for visualizing the data.

Molecular docking was performed between the top 5 core targets ranked by decreasing degree values and the selected key components. The 3D structures of key active components of drugs were downloaded from the PubChem database (https://pubchem.ncbi.nlm.nih.gov/), and the high-resolution 3D crystal structures of core target proteins were downloaded from the RCSB PDB database. The 3D structures of core targets were optimized using the CB-Dock2 website (https://cadd.labshare.cn/cb-dock2/php/index.php) by removing the original ligands, removing water molecules, separating proteins, and adding hydrogen atoms. The binding activity of components to targets was evaluated using binding energy. The larger the negative binding energy, the more stable the docking conformation and the stronger the spontaneous binding ability. The most stable conformation was selected, and the molecular docking visualization results were downloaded for analysis.

#### 4.2.3. Pharmacological Experimental Methods

The animal experiments were conducted in accordance with the National Act on the Use of Experimental Animals (China) and approved by the Academic Committee of Southwest Minzu University (No.: SMU-202401114).

According to Feng et al. [27], corynoline exhibits excellent hepatoprotective effects against acute liver injury induced by carbon tetrachloride in mice. The ACE obtained in this study is a derivative of corynoline, and it is speculated to possess similar biological activities. Therefore, the dosage was referenced from this study. To facilitate the comparison of the biological activity of various compounds (ACE, BBC, ISO), the same high, medium, and low doses were used for different alkaloid components. Meanwhile, we have taken into account the inherent toxicity of the compound. According to literature reports, the median lethal doses (LD50) for ISO via tail vein injection and intraperitoneal injection in mice were 49 mg/kg and 52 mg/kg, respectively. The doses used in this experiment are significantly lower than these values [28]. However, due to the complexity of the TTA components and the relatively low content of each alkaloid, the dosage was increased tenfold.

Eighty healthy male KM mice (weighing 20 ± 2 g) were randomly divided into 15 groups, with five mice in each group. These included the CON, CCl_4_, SIL (10 mg/kg), TTA (50, 100, and 200 mg/kg), and three drug treatment groups (ACE, BBC, and ISO) at high, medium, and low doses (5, 10, and 20 mg/kg). After a week of normal feeding in the mouse room, the mice were administered drugs. The SIL, TTA, ACE, BBC, and ISO groups were gavaged with the predetermined doses daily for 14 days, while the CON and CCl_4_ groups were gavaged with saline. After the last administration, the CON group was gavaged with olive oil solvent without CCl_4_, while the remaining groups were gavaged with 0.2% CCl_4_ in olive oil solution for modeling. After gavage, the mice were fasted for 24 h without water restriction. After fasting, the eyeballs of the mice were removed for blood collection. The whole blood was centrifuged at 4000 r/min for 10 min, and the upper serum layer was stored at −20 °C for serum biochemical parameter detection. After serum sampling, the liver lobules of mice in each group were excised and fixed in 4% paraformaldehyde for routine histological examination and frozen sectioning. The remaining liver tissues were stored in a −80 °C refrigerator.

The serum was frozen at −20 °C, and the levels of ALT, AST, and ALP in the serum were measured according to the instructions provided in the reagent kit.

An amount of 0.5 g of the same part of the liver was accurately weighed, and a liver homogenate was prepared with physiological saline at a ratio of 1:9 (m/V). The CRP content in the liver tissue was measured according to the instructions provided in the reagent kit.

The resected left liver lobe specimen was fixed with 4% paraformaldehyde. The specimen was dehydrated using an automatic machine. The parameters for each step were as follows: 70% EtOH for 4 h, 80% EtOH for 2 h, 90% EtOH for 2 h, 95% EtOH for 2 h, 2 portions of anhydrous EtOH for 1 h each, 2 portions of xylene for 30 min each, 3 portions of paraffin, and 60 °C for 1.5 h each. Conventional rotary paraffin sections were made with a thickness of 4 μm. The sections were dewaxed with xylene, stained with conventional hematoxylin–eosin, dehydrated, cleared, and mounted with neutral gum. The sections were scanned using a 3DHISTECH (Hungary) Pannoramic SCAN digital slide scanner for image acquisition. After observation and analysis, specific magnifications of microscopic images were captured, and low- or high-magnification images were taken for comparison and explanation.

Data were statistically analyzed using SPSS 10.0 software and Origin 8.0 for image processing and are expressed as the mean ± standard deviation. The *t*-test was used for comparisons between groups, and ANOVA was applied for comparisons of multiple group means. Significant differences were indicated when *p* < 0.05.

## 5. Conclusions

A chemical investigation of the whole herb of the Tibetan medicine *Corydalis conspersa* Maxim. resulted in the isolation and purification of seven alkaloids: acetylcorynoline (**1**), corynoline (**2**), scoulerine (**3**), protopine (**4**), bulbocapnine (**5**), palmatine (**6**), and isocorydine (**7**). The structures of these compounds were elucidated using NMR spectroscopy and by comparison with literature data. Among them, compounds 1, 5, and 7 were isolated for the first time from *Corydalis conspersa* Maxim. Furthermore, pharmacological experiments have shown that compounds **1**, **5**, **7**, and TTA have therapeutic effects on acute liver injury induced by carbon tetrachloride in mice, and the therapeutic effect is dose-dependent. NP predicted that the mechanism by which these alkaloids exert hepatoprotective activity may be through STAT3, SRC, EGFR, PIK3CA, HSP90AA1, and other targets that exert their effects.

In summary, the Tibetan medicine *Corydalis conspersa* Maxim. has been shown to be a good source of bioactive alkaloids. However, the molecular mechanisms have not been thoroughly explored; The ALI model induced solely by CCl_4_ needs to be extended to other models (such as alcoholic liver injury) to verify its universality. The research team will continue to explore its mechanism of action in subsequent experiments.

## Figures and Tables

**Table 1:** Five core targets and degree values.

Order Number	Gene	Degree
1	*STAT3*	30
2	*EGFR*	29
3	*HSP90AA1*	25
4	*ESR1*	23
5	*PIK3CA*	20

**Table 2:** The top five core active ingredients ranked by degree values.

Number	Name	CAS or Pubchem ID	Degree
1	Bicuculline	485-49-4	43
2	Acetylcorynoline	18797-80-3	30
3	Corydaline	6451-73-6	25
4	Bulbocapnine	298-45-3	24
5	Isocorydine	475-67-2	24

**Table 3:** Changes in AST, ALT, and ALP levels in mouse serum (mean ± *SD*, *n* = 5/group).

Groups	AST/(U·L^−1^)	ALT/(U·L^−1^)	ALP/(U·L^−1^)
CON	132.43 ± 12.06	42.80 ± 7.18	56.60 ± 5.51
CCl_4_	405.50 ± 18.34 ^###^	205.60 ± 13.93 ^###^	255.23 ± 12.44 ^###^
ACE 5 mg/kg	331.93 ± 15.95 ***	169.97 ± 11.51 **	223.43 ± 14.54 **
ACE 10 mg/kg	279.23 ± 12.58 ***	131.30 ± 12.35 ***	175.20 ± 12.87 ***
ACE 20 mg/kg	184.07 ± 14.45 ***	91.23 ± 6.09 ***	92.08 ± 10.06 ***
BBC 5 mg/kg	316.23 ± 12.55 ***	173.13 ± 10.27 **	217.87 ± 13.08 ***
BBC 10 mg/kg	246.93 ± 9.11 ***	149.60 ± 8.22 ***	151.60 ± 10.98 ***
BBC 20 mg/kg	169.63 ± 18.23 ***	101.50 ± 12.46 ***	104.07 ± 8.67 ***
ISO 5 mg/kg	345.64 ± 13.31 ***	178.61 ± 9.72 **	238.52 ± 10.29 *
ISO 10 mg/kg	291.34 ± 10.72 ***	152.93 ± 12.18 ***	189.97 ± 11.73 ***
ISO 20 mg/kg	223.58 ± 16.29 ***	126.27 ± 11.24 ***	131.82 ± 8.18 ***
TTA 50 mg/kg	326.81 ± 11.84 ***	164.59 ± 9.41 **	229.39 ± 9.92 **
TTA 100 mg/kg	261.39 ± 13.05 ***	138.67 ± 8.08 ***	162.21 ± 8.06 ***
TTA 200 mg/kg	208.67 ± 9.12 ***	93.25 ± 12.57 ***	110.41 ± 6.27 ***
SIL 10 mg/kg	167.28 ± 16.17 ***	83.18 ± 8.29 ***	112.26 ± 7.69 ***

Note: ^###^ *p* < 0.001, compared with CON group; * *p* < 0.05, ** *p* < 0.01, *** *p* < 0.001, compared with CCl_4_ group.

**Table 4:** Changes in CRP levels in mouse liver (mean ± *SD*, *n* = 5/group).

Groups	CRP/ (μg·L^−1^)
CON	541.93 ± 34.84
CCl_4_	1792.50 ± 147.36 ^###^
ACE 5 mg/kg	1509.87 ± 136.31 *
ACE 10 mg/kg	1183.27 ± 104.47 ***
ACE 20 mg/kg	729.20 ± 112.17 ***
BBC 5 mg/kg	1558.87 ± 134.30 *
BBC 10 mg/kg	1241.40 ± 121.32 ***
BBC 20 mg/kg	796.53 ± 91.47 ***
ISO 5 mg/kg	1631.92 ± 135.68
ISO 10 mg/kg	1425.64 ± 118.29 **
ISO 20 mg/kg	947.56 ± 124.64 ***
TTA 50 mg/kg	1438.81 ± 141.21 **
TTA 100 mg/kg	1221.34 ± 128.63 ***
TTA 200 mg/kg	803.91 ± 99.71 ***
SIL 10 mg/kg	857.81 ± 81.82 ***

Note: ^###^ *p* < 0.001 compared with CON group; * *p* < 0.05, ** *p* < 0.01, and *** *p* < 0.001 compared with CCl_4_ group.

## Data Availability

The original contributions presented in this study are included in the article/Supplementary Materials.

## Supplementary Materials

The following supporting information can be downloaded at: https://www.mdpi.com/article/10.3390/molecules30102127/s1. 1H-NMR, 13C-NMR and HR-ESI-MS for acetylcorynoline (**1**, Figures S1–S3); 1H-NMR, 13C-NMR and HR-ESI-MS for corynoline (**2**, Figures S4–S6); 1H-NMR, 13C-NMR and HR-ESI-MS for scoulerine (**3**, Figures S7–S9); 1H-NMR, 13C-NMR and HR-ESI-MS for protopine (**4**, Figures S10–S12); 1H-NMR, 13C-NMR and HR-ESI-MS for bulbocapine (**5**, Figures S13–S15); 1H-NMR, 13C-NMR and HR-ESI-MS for palmatine (**6**, Figures S16–S18); 1H-NMR, 13C-NMR and HR-ESI-MS for isocorydine (**7**, Figures S19–S21).

## Abbreviations

The following abbreviations are used in this manuscript:

**Abbreviation**	**Full name of compound**
CCl_4_	Carbon tetrachloride
ALI	Acute liver injury
NP	Network pharmacology
ACE	Acetylcorynoline
BBC	Bulbocorynoline
ISO	Isocorynidine
KM	Kunming
PPI	Protein–Protein Interaction
GO	Gene ontology
KEGG	Kyoto Encyclopedia of Genes and Genomes
NP	Network pharmacology
BP	Biological process
CC	Cellular component
MF	Molecular function
AST	Aspartate aminotransferase
ALT	Alanine aminotransferase
ALP	Alkaline phosphatase
CON	Control
SIL	Silymarin
CRP	C-reactive protein
MeOH	Methanol
PE	Petroleum ether
EtOAc	Ethyl acetate
CHCl_3_	Chloroform
HCl	Hydrochloric acid
EtOH	Anhydrous ethanol
SGCC	Silica gel column chromatography
LPS	Lipopolysaccharide
D-GaIN	D-Galactosamine
