# Supplementary material for: Alkaloids in Tibetan Medicine Corydalis conspersa Maxim. and Their Hepatoprotective Effect Against Acute Liver Injury
Source: Molecules. 2025 May 11;30(10):2127. doi: 10.3390/molecules30102127 (PMC12114063; doi:10.3390/molecules30102127)
Supplement: Supplementary file 1 [file molecules-30-02127-s001.zip › molecules-3599546-supplementary.pdf]

Alkaloids in Tibetan Medicine *Corydalis conspersa* Maxim. and Their Hepatoprotective Effect Against Acute Liver Injury

Qiu Wang, Yingrui Jin, Fangyan Fan, Xueting Feng, Xuemei Yin, Xiaoling Wang * and Zangjia Geng *

College of Pharmacy and Food, Southwest Minzu University, Chengdu 610041, China; 221008002019@stu.swun.edu.cn (Q.W.); 231008002025@stu.swun.edu.cn (Y.J.); 202131803011@stu.swun.edu.cn (F.F.) 80300225@swun.edu.cn (X.F.); xuemeiyin@swun.edu.cn (X.Y.)

* Correspondence: 21900034@swun.edu.cn (X.W.); 21800007@swun.edu.cn (Z.G.)

Compound **Page**

^{1}H-NMR , ^{13}C-NMR and HR-ESI-MS for acetylcorynoline (1)........ 2

^{1}H-NMR , ^{13}C-NMR and HR-ESI-MS for corynoline (2)........ 3

^{1}H-NMR , ^{13}C-NMR and HR-ESI-MS for scoulerine (3) 5

^{1}H-NMR , ^{13}C-NMR and HR-ESI-MS for protopine (4) 6

^{1}H-NMR , ^{13}C-NMR and HR-ESI-MS for bulbocapine (5)........ 8

^{1}H-NMR , ^{13}C-NMR and HR-ESI-MS for palmatine (6)........ 9

^{1}H-NMR , ^{13}C-NMR and HR-ESI-MS for isocorydine (7)........ 11

^{1}H-NMR , ^{13}C-NMR and HR-ESI-MS for acetylcorynoline (1)

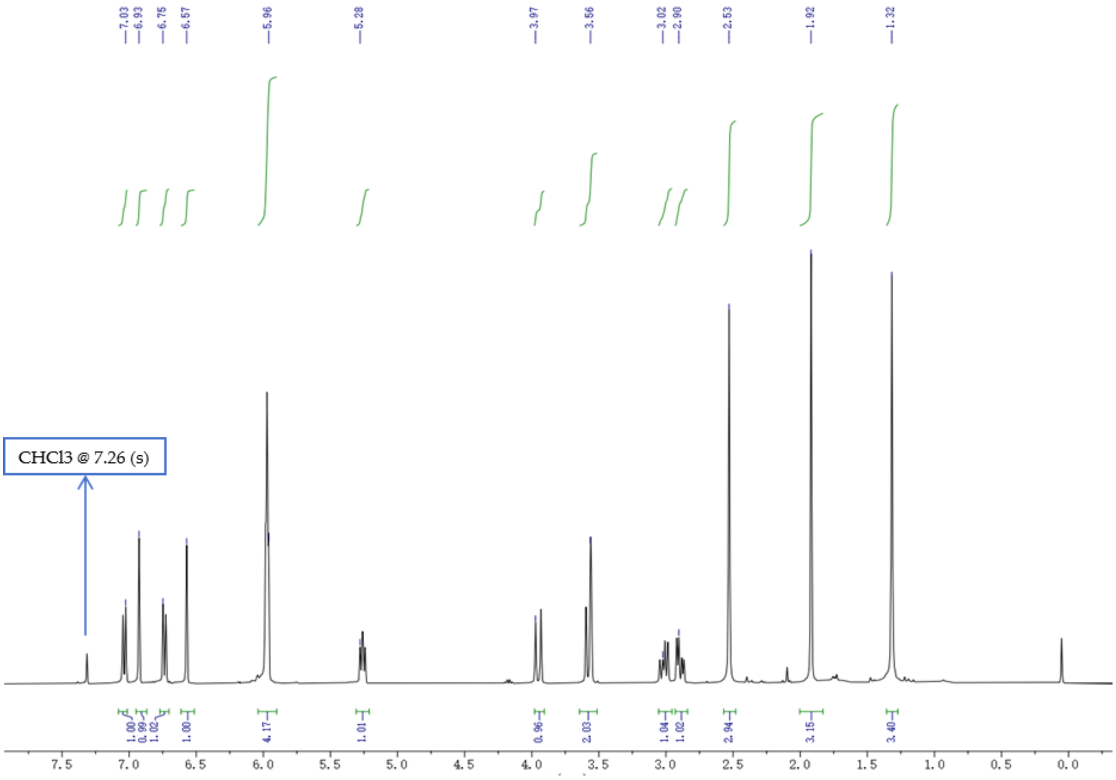

Figure S1. ^{1}H-NMR for acetylcorynoline.

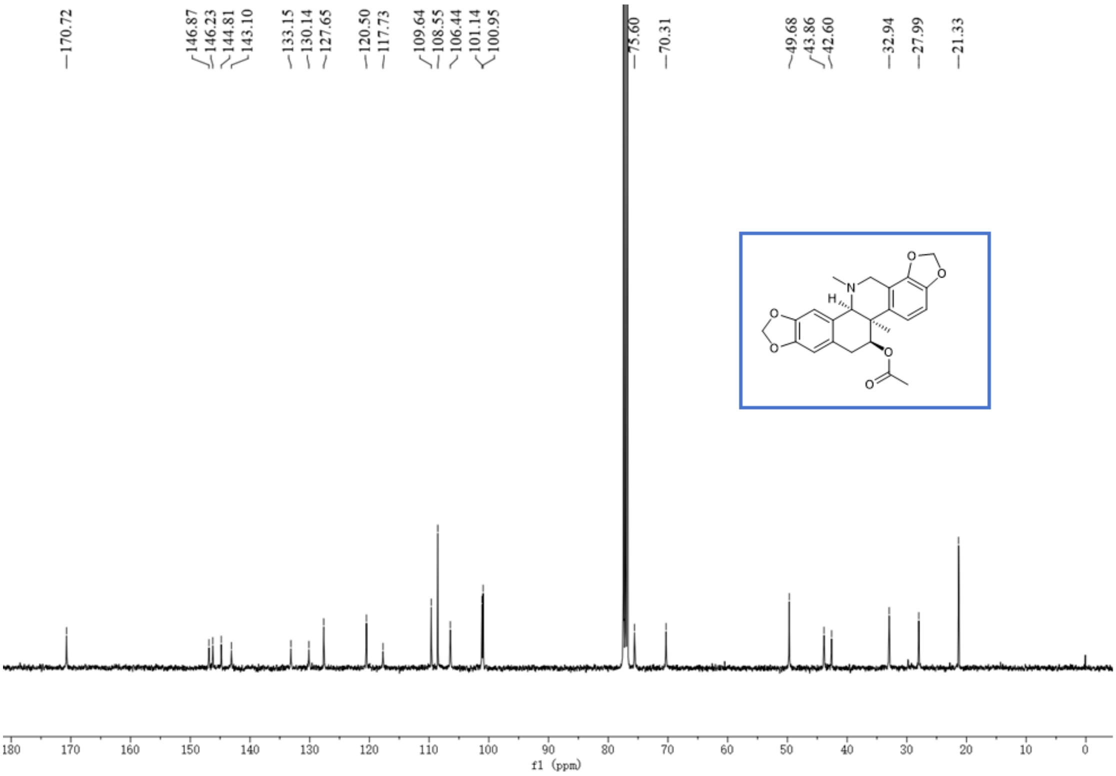

Figure S2. ^{13}C-NMR for acetylcorynoline.

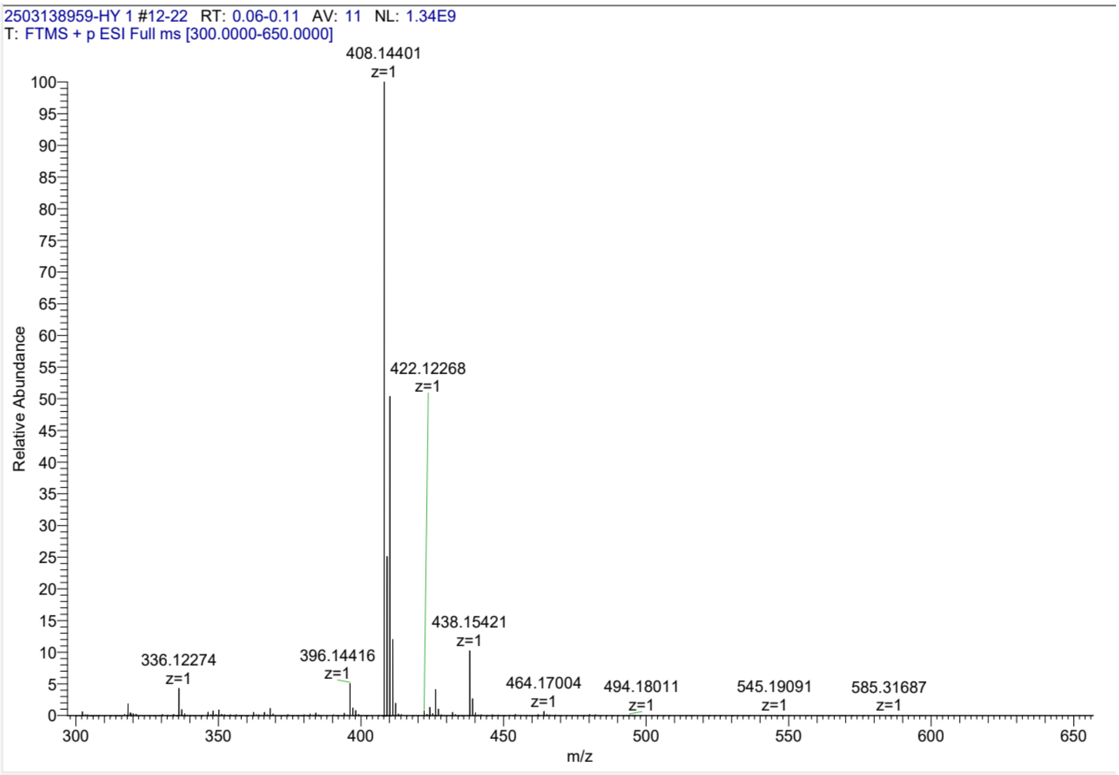

Figure S3. HR-ESI-MS for acetylcorynoline.

¹H-NMR , ¹³C-NMR and HR-ESI-MS for corynoline (2)

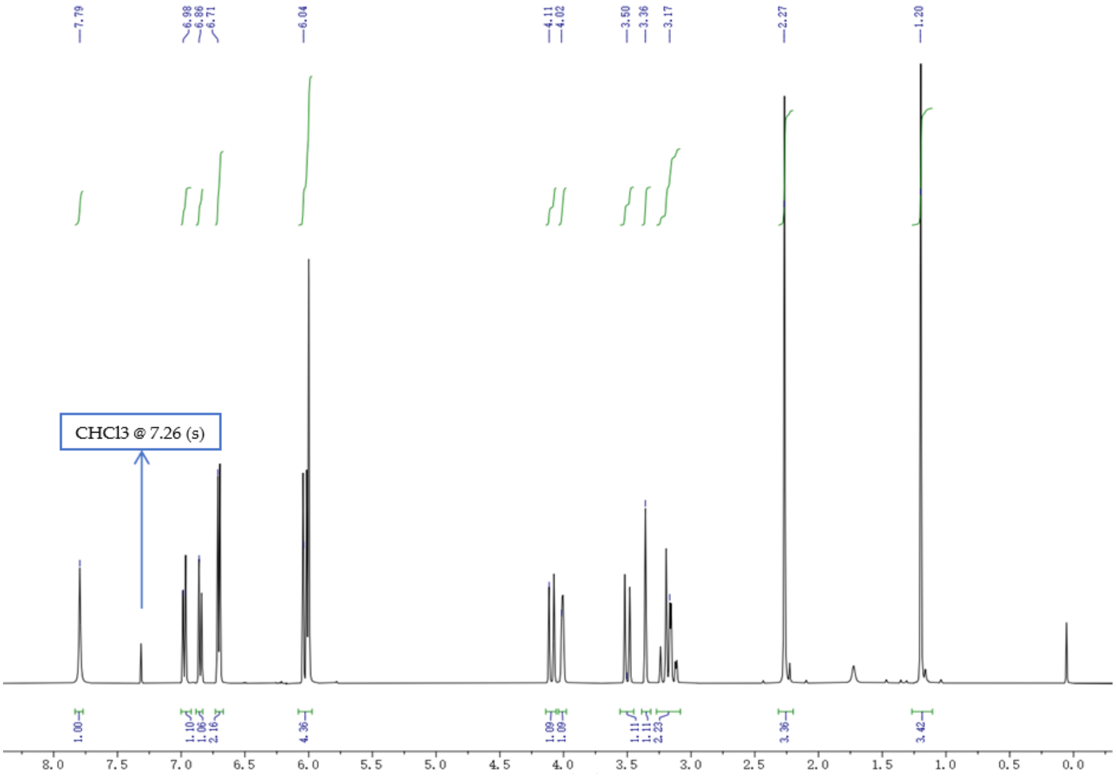

Figure S4. ¹H-NMR for corynoline.

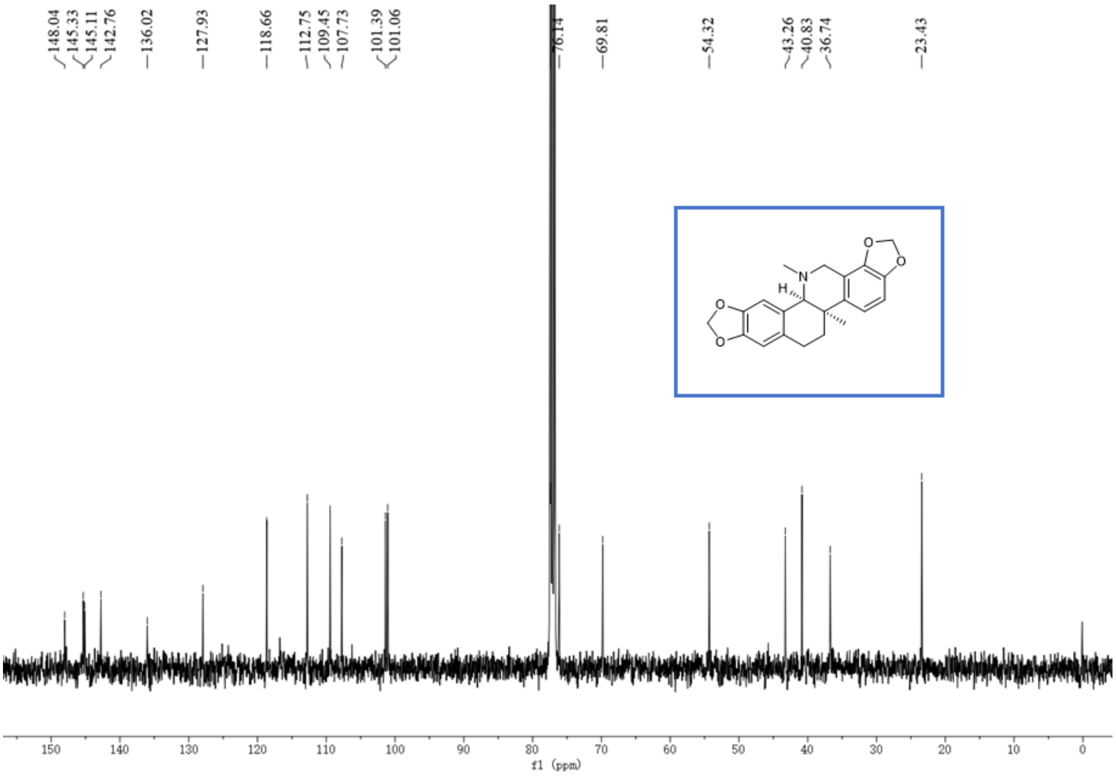

Figure S5. ^{13}C-NMR for corynoline.

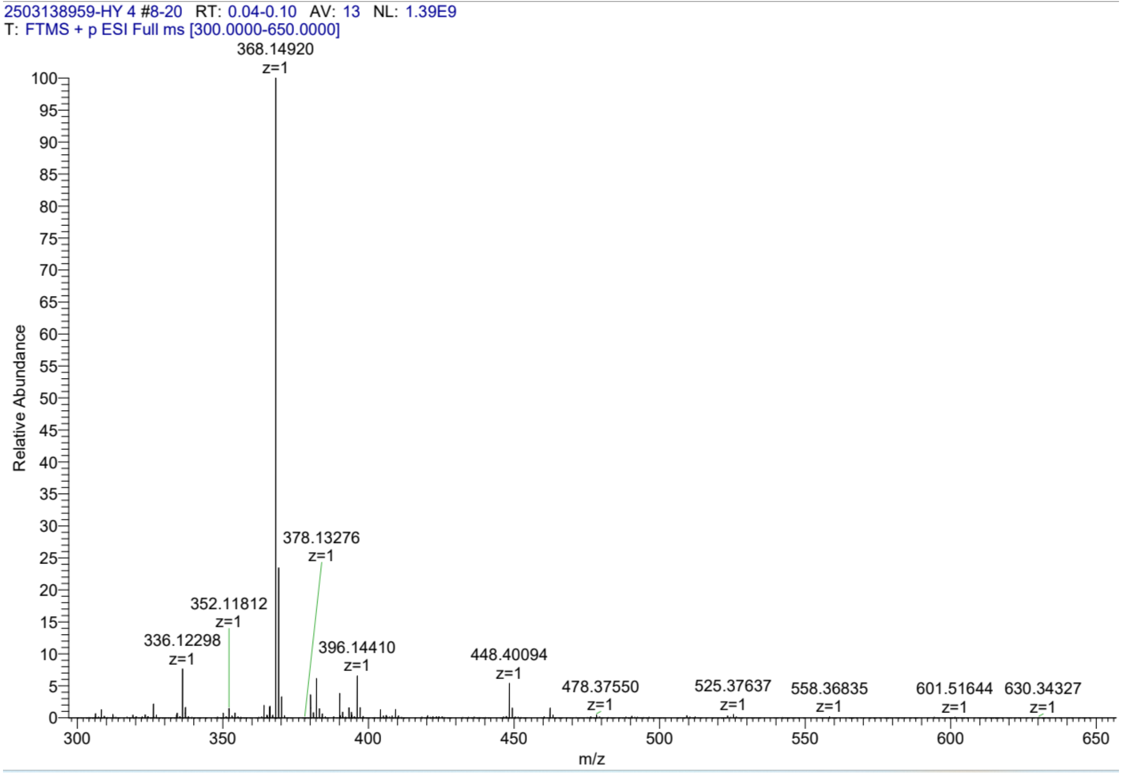

Figure S6. HR-ESI-MS for corynoline.

¹H-NMR , ¹³C-NMR and HR-ESI-MS for scoulerine (3)

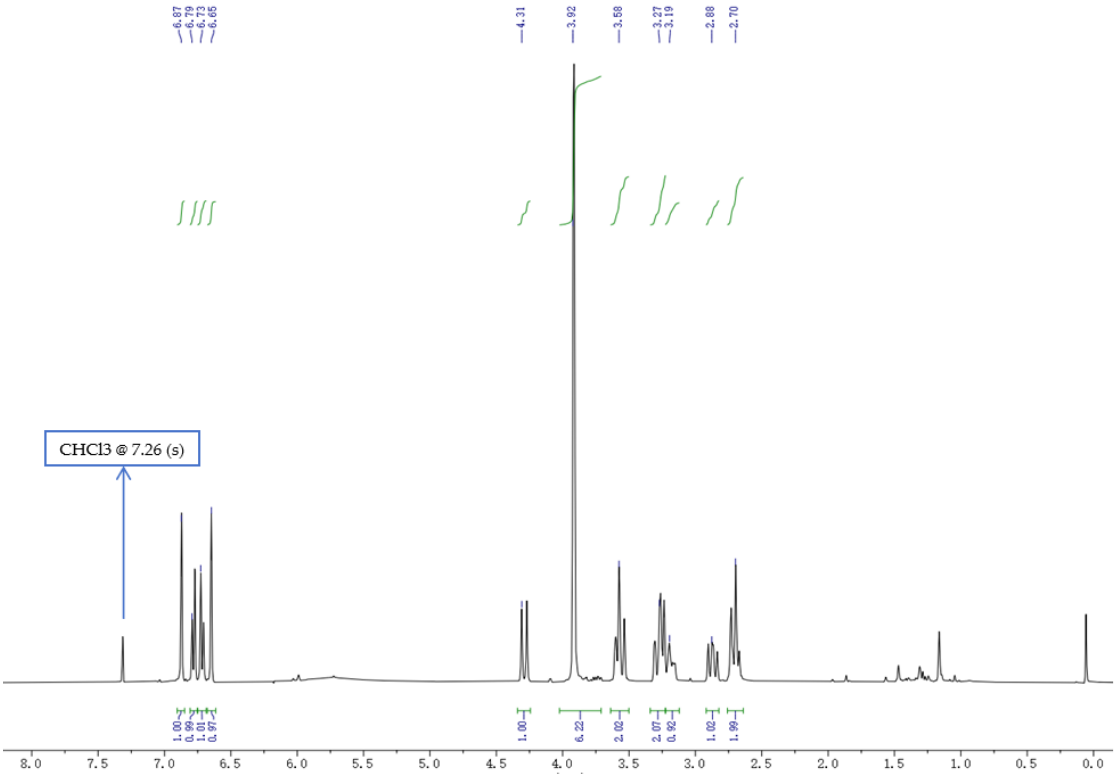

Figure S7. ^{1}H-NMR for scoulerine.

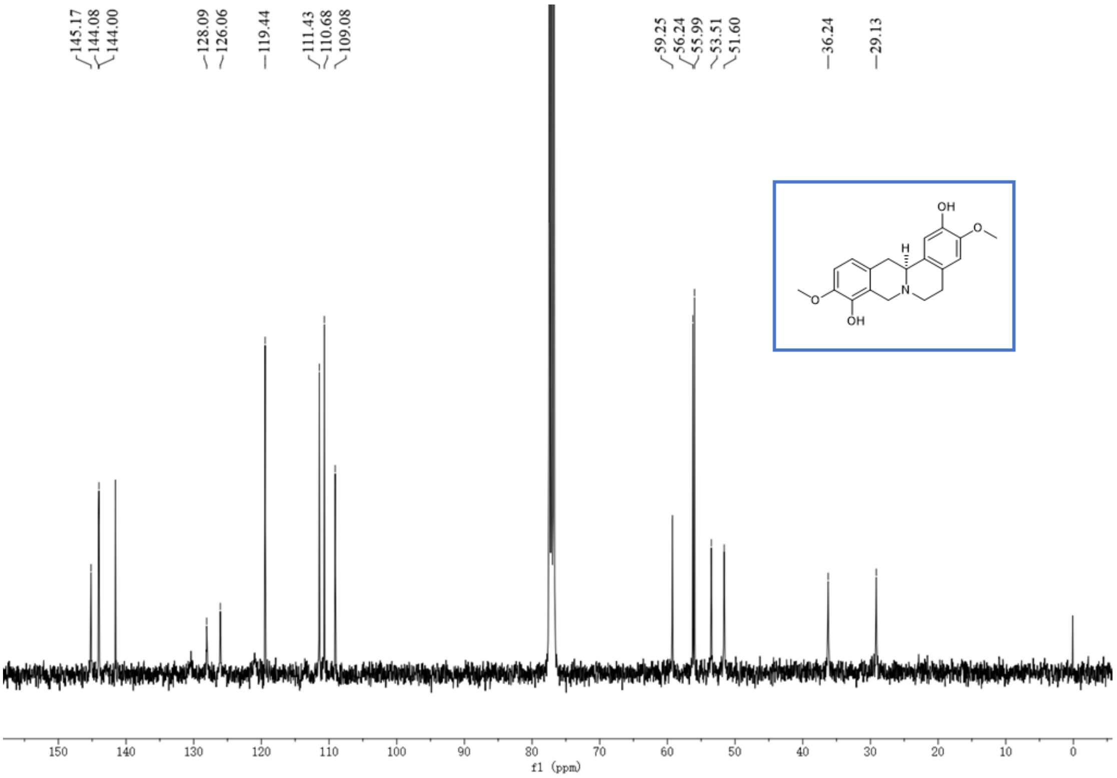

Figure S8. ^{13}C-NMR for scoulerine.

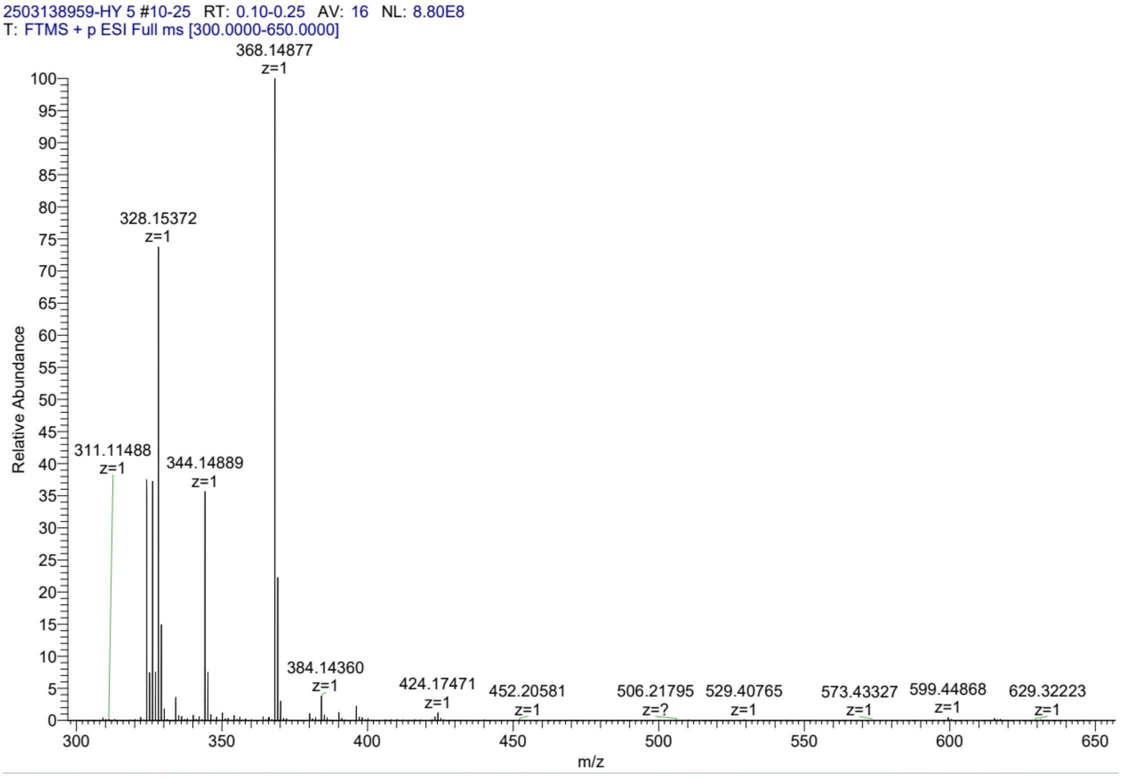

Figure S9. HR-ESI-MS for scoulerine.

^{1}H-NMR , ^{13}C-NMR and HR-ESI-MS for protopine (4)

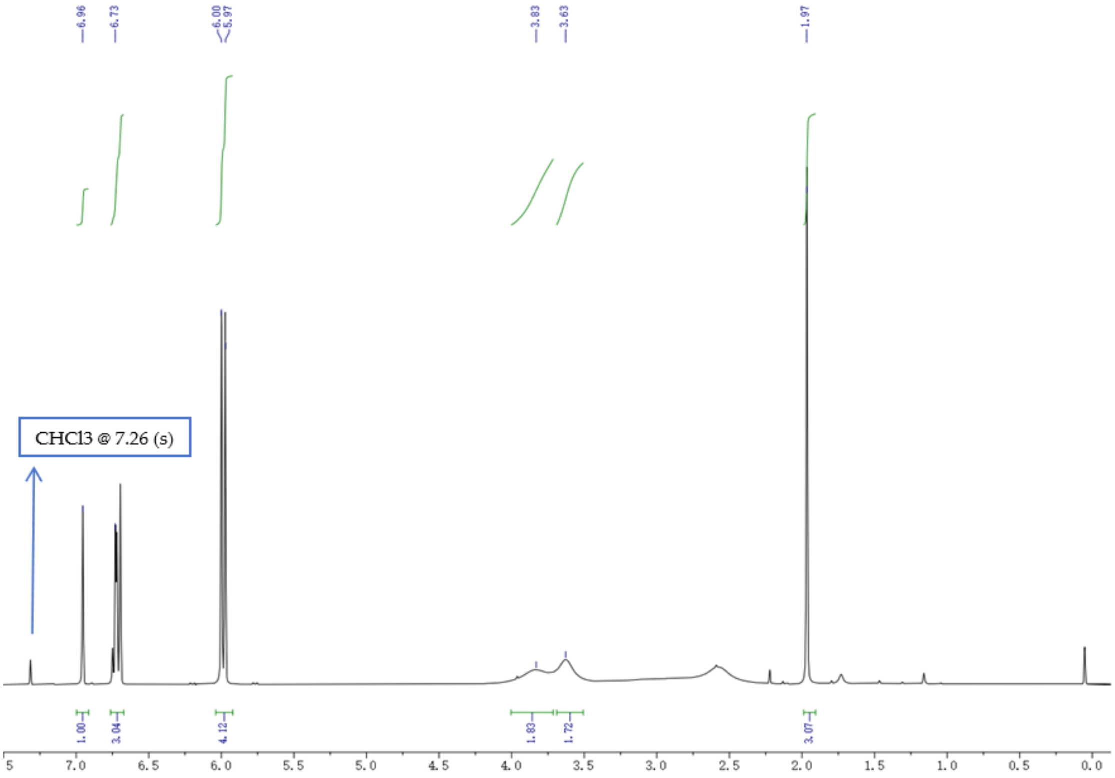

Figure S10. ^{1}H-NMR for protopine.

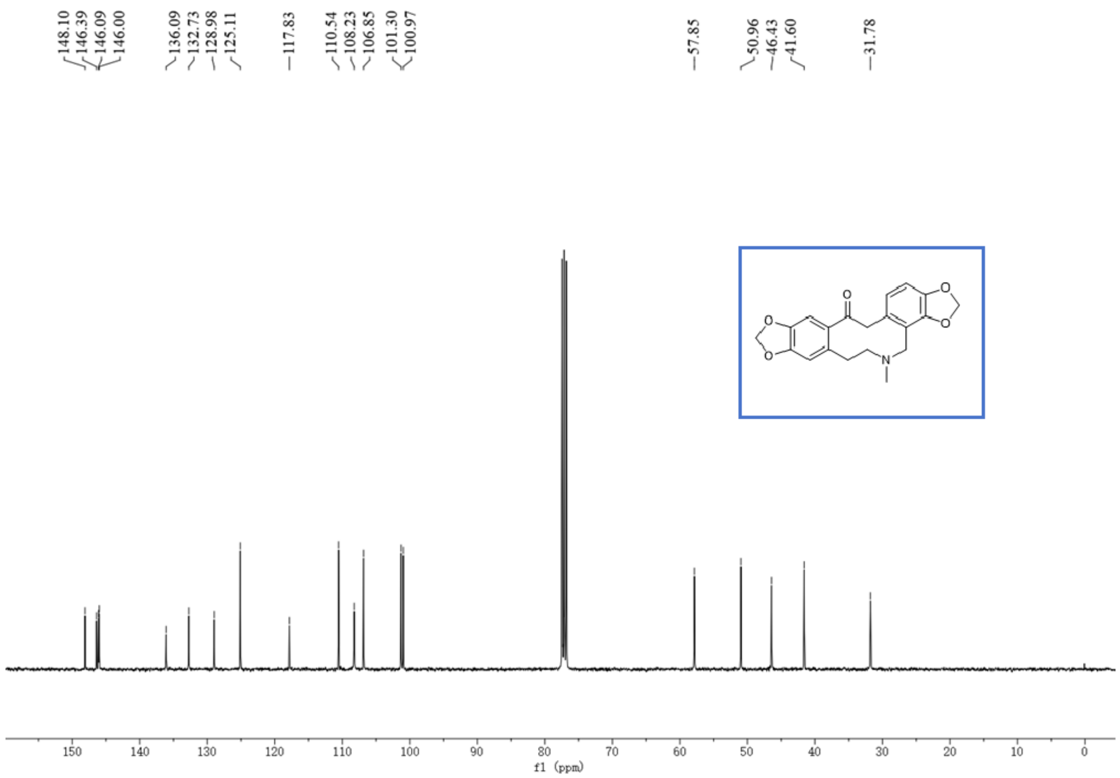

Figure S11. ^{13}C-NMR for protopine.

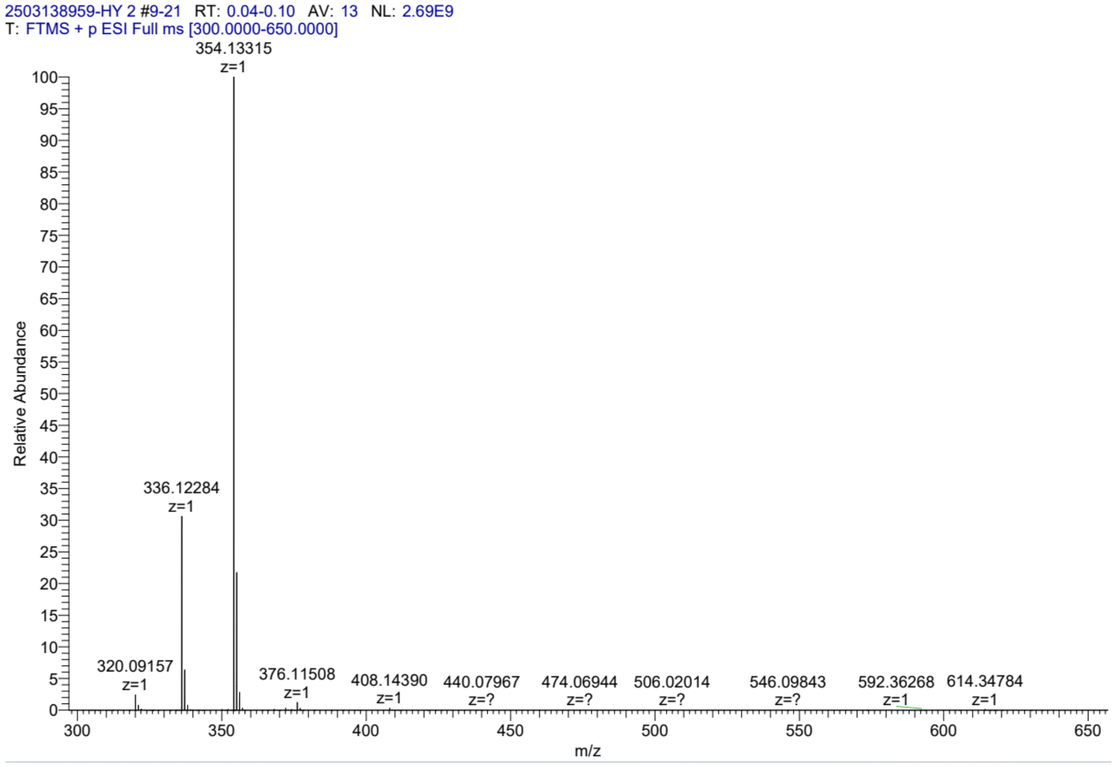

Figure S12. HR-ESI-MS for protopine.

^{1}H-NMR , ^{13}C-NMR and HR-ESI-MS for bulbocapine (5)

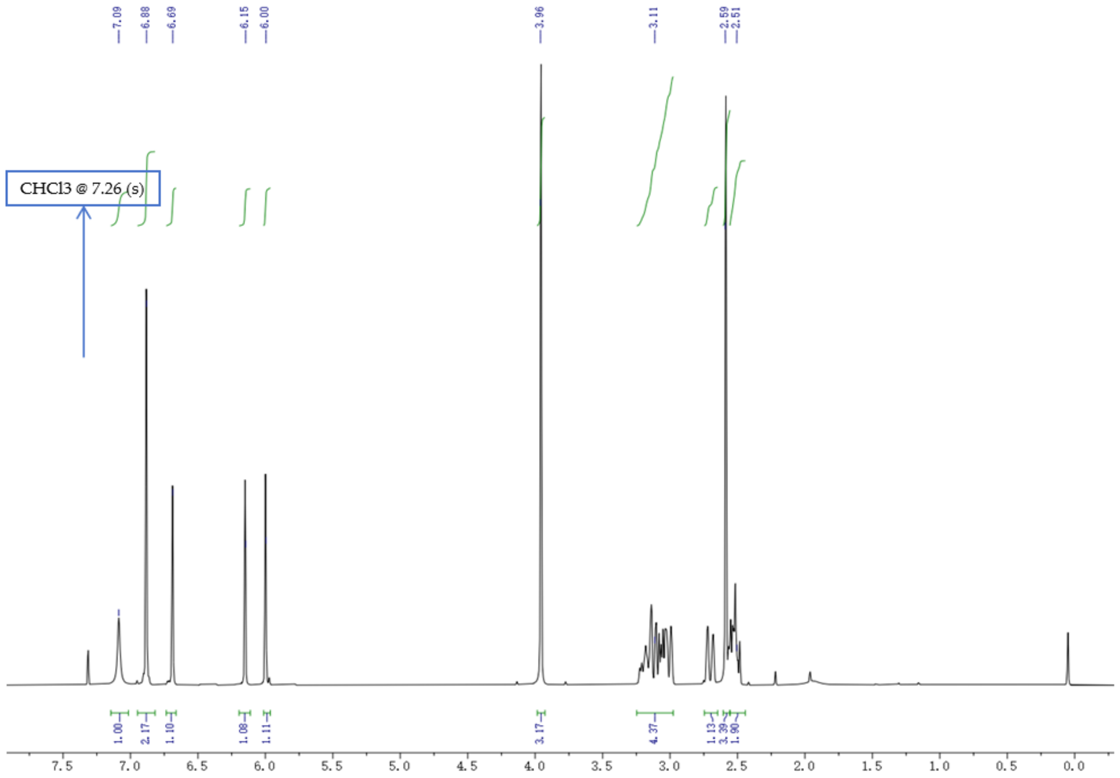

Figure S13. ^{1}H-NMR for bulbocapine.

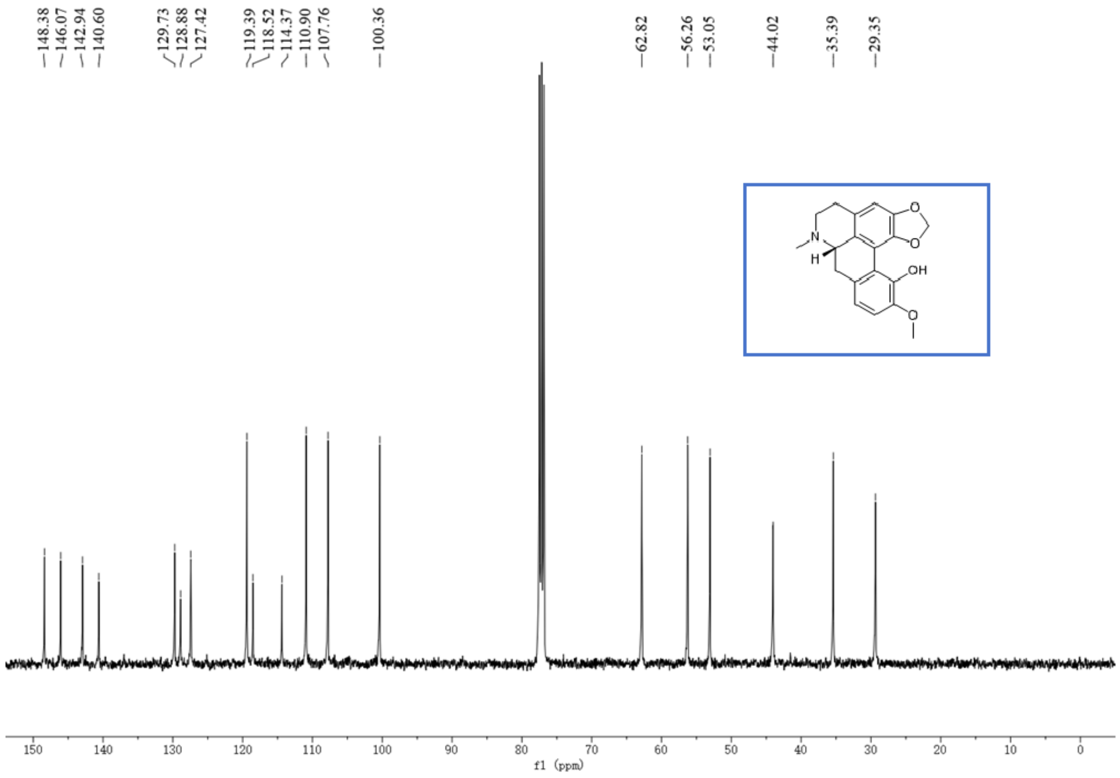

Figure S14. ^{13}C-NMR for bulbocapine.

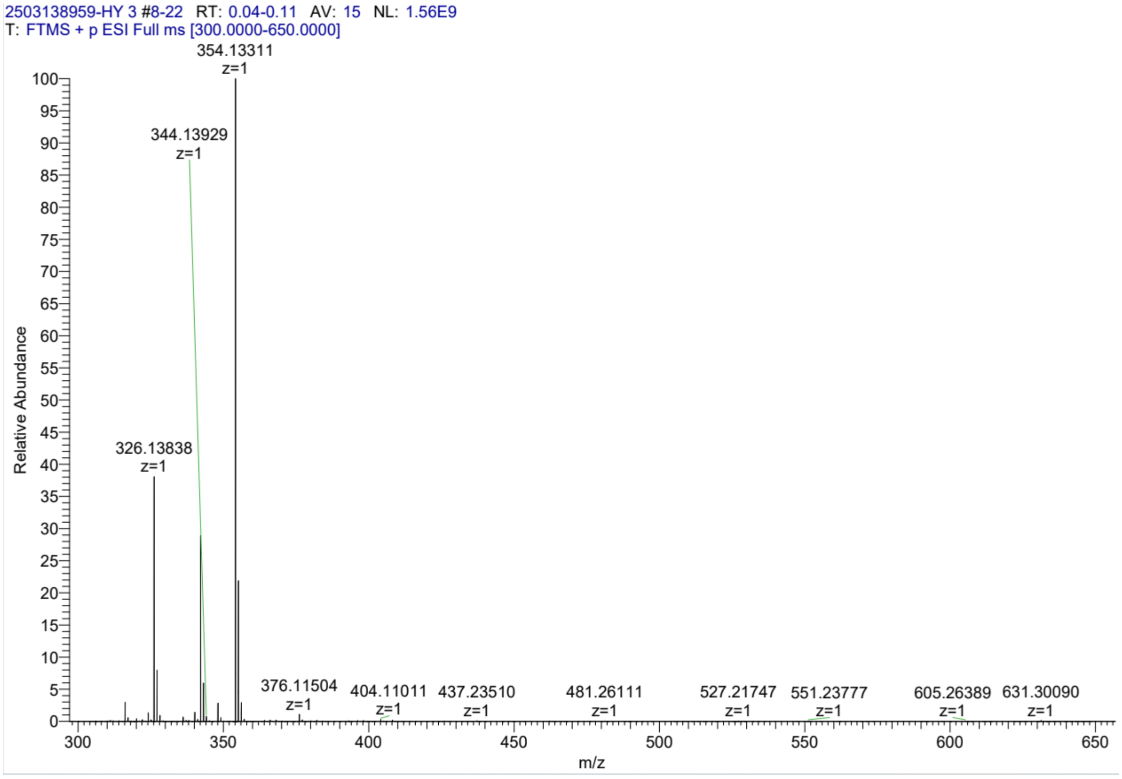

Figure S15. HR-ESI-MS for bulbocapine.

^{1}H-NMR , ^{13}C-NMR and HR-ESI-MS for palmatine (6)

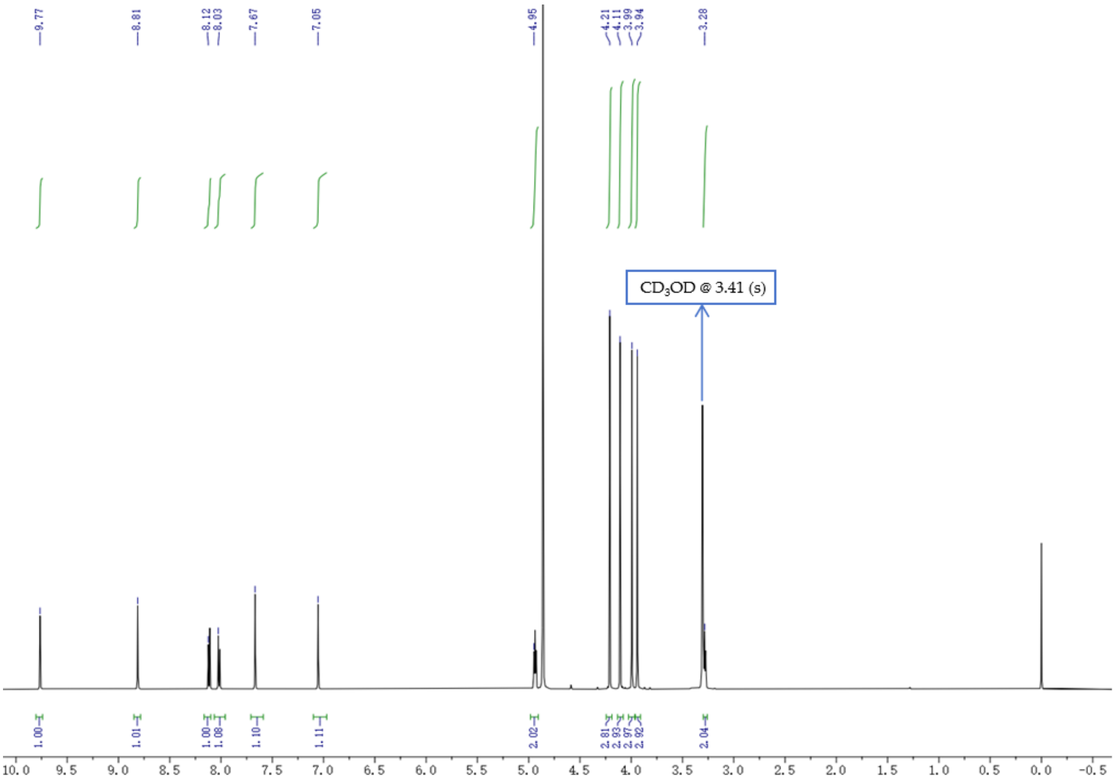

Figure S16. ^{1}H-NMR for palmatine.

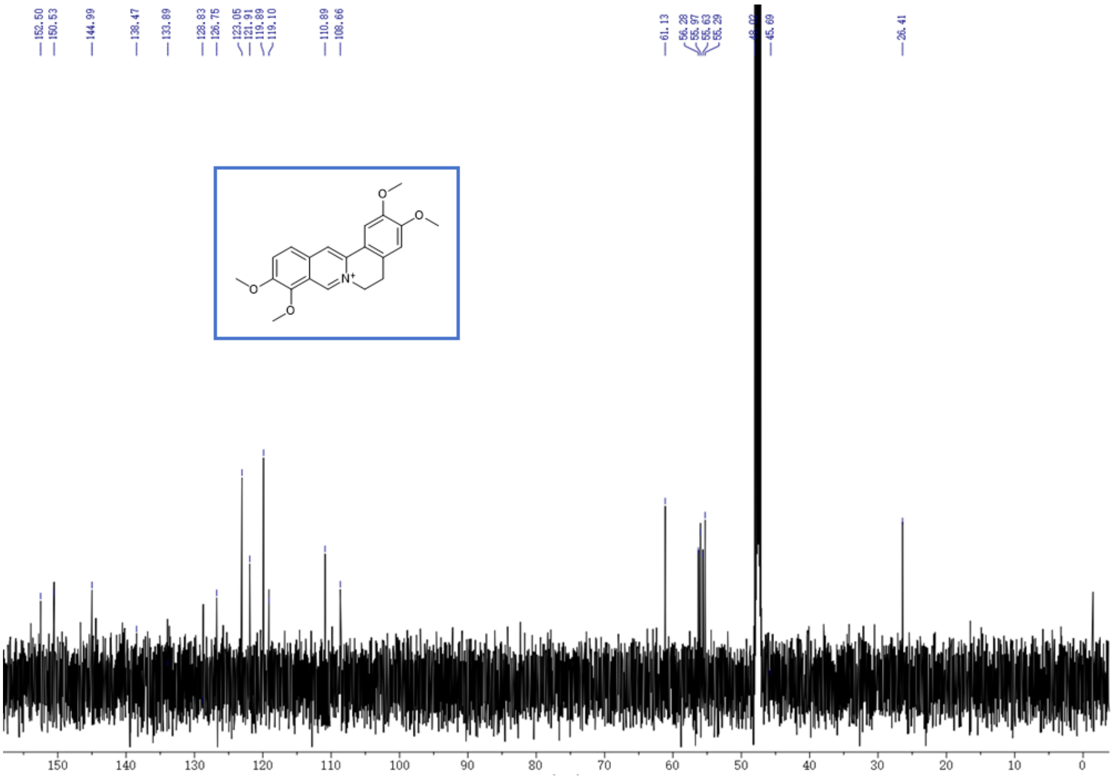

Figure S17. ^{13}C-NMR for palmatine.

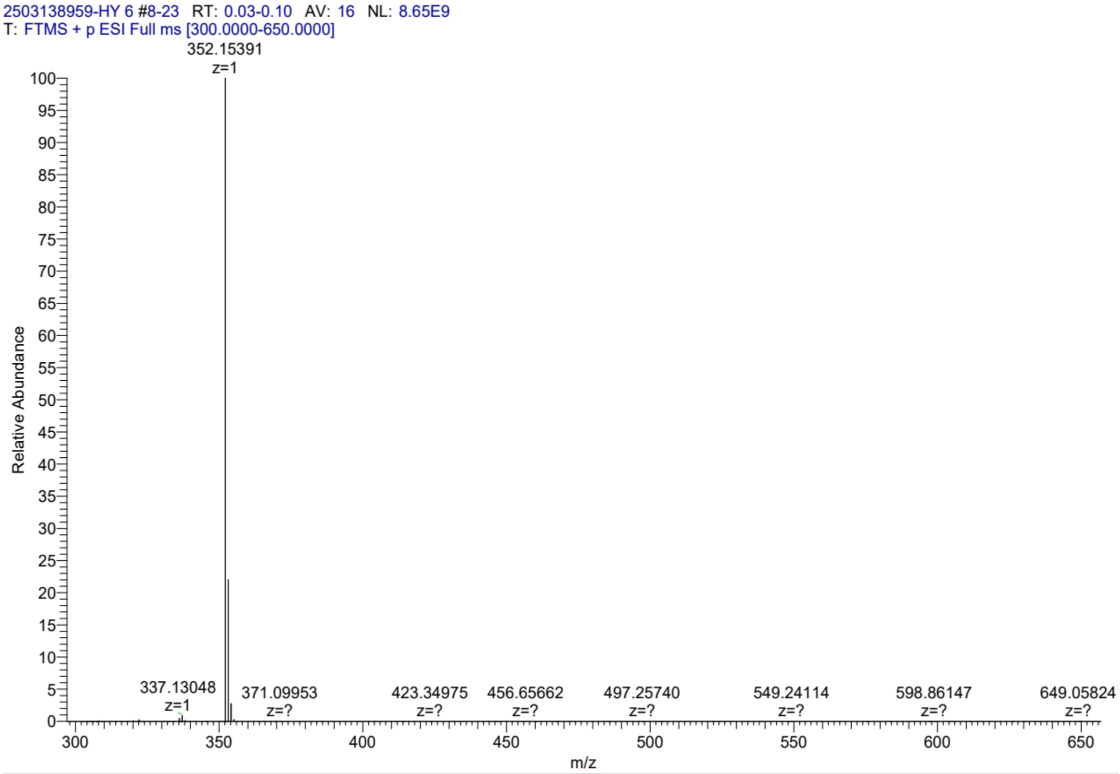

Figure S18. HR-ESI-MS for palmatine.

^{1}H-NMR , ^{13}C-NMR and HR-ESI-MS for isocorydine (7)

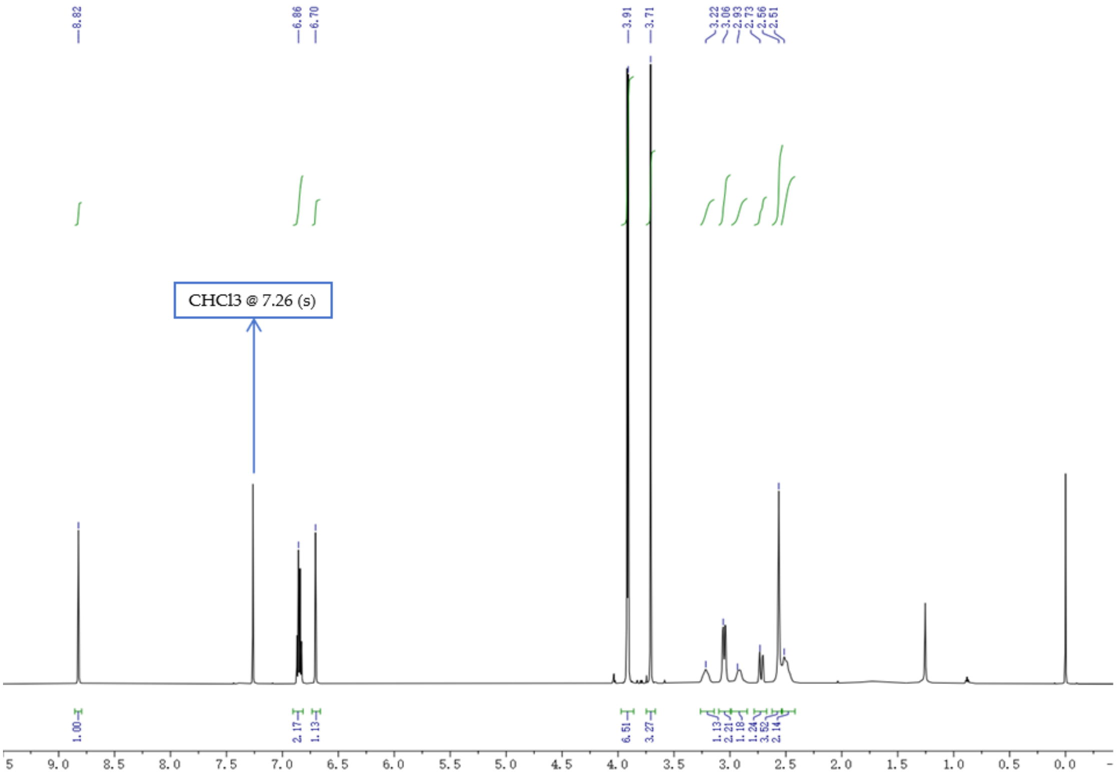

Figure S19. ^{1}H-NMR for isocorydine.

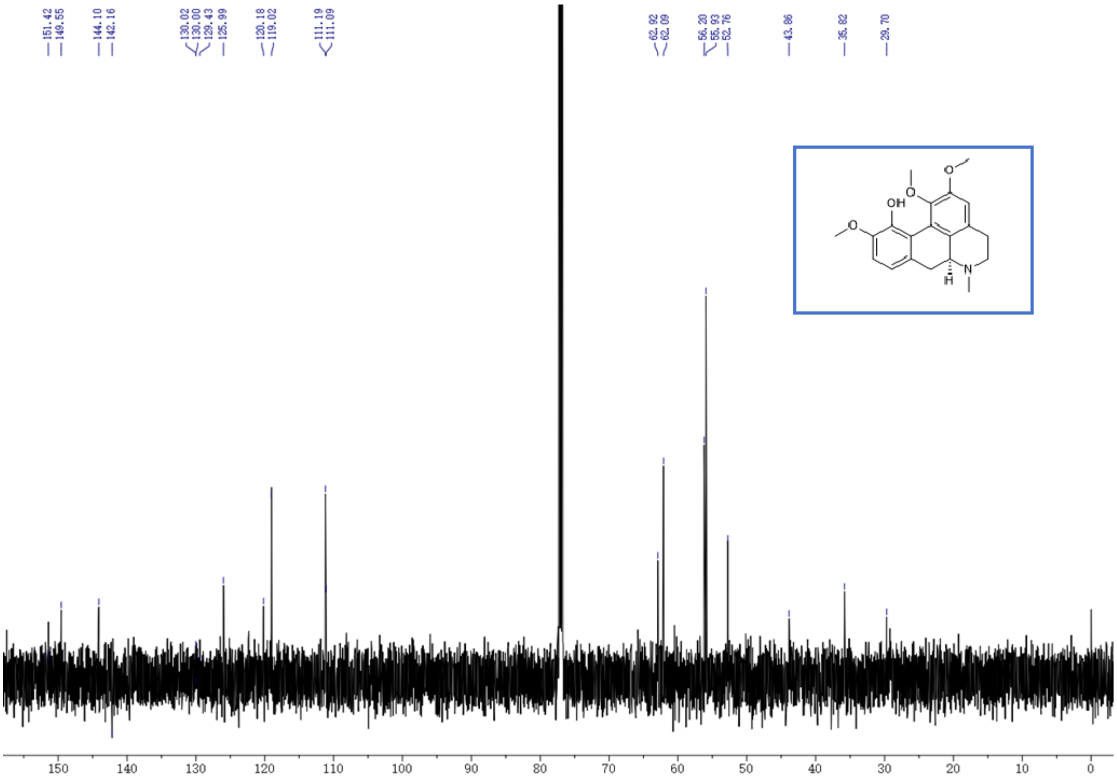

Figure S20. ^{13}C-NMR for isocorydine.

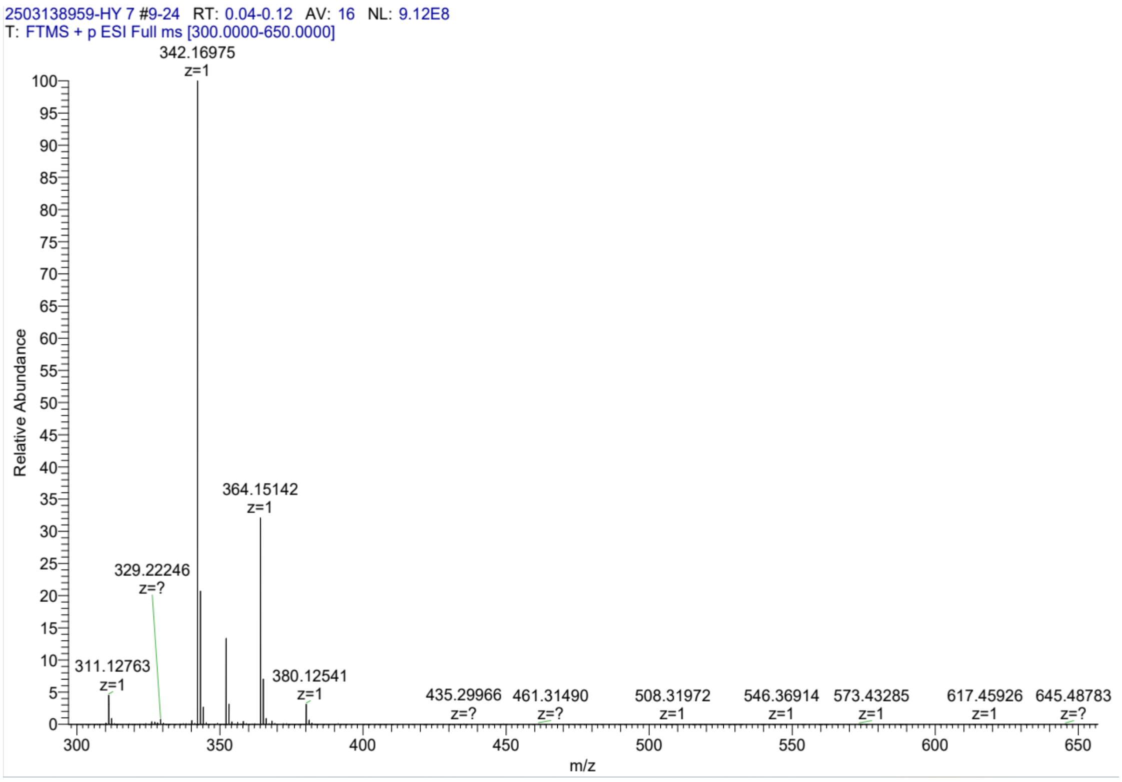

Figure S21. HR-ESI-MS for isocorydine.
